# Genetic Interaction Between Site-Specific Epigenetic Marks and Roles of H4v in Transcription Termination in *Trypanosoma brucei*

**DOI:** 10.3389/fcell.2021.744878

**Published:** 2021-10-14

**Authors:** Hee-Sook Kim

**Affiliations:** Public Health Research Institute, New Jersey Medical School, Rutgers, The State University of New Jersey, Newark, NJ, United States

**Keywords:** *Trypanosoma brucei*, transcription termination site, chromatin marks, DNA replication, cell-cycle progression, chromosome segregation, monoallelic VSG expression

## Abstract

In *Trypanosoma brucei*, genes are assembled in polycistronic transcription units (PTUs). Boundaries of PTUs are designated transcription start sites and transcription termination sites (TTSs). Messenger RNAs are generated by *trans*-splicing and polyadenylation of precursor RNAs, and regulatory information in the 3′ un-translated region (UTR), rather than promoter activity/sequence-specific transcription factors, controls mRNA levels. Given this peculiar genome structure, special strategies must be utilized to control transcription in *T. brucei*. TTSs are deposition sites for three non-essential chromatin factors—two of non-canonical histone variants (H3v and H4v) and a DNA modification (base J, which is a hydroxyl-glucosyl dT). This association generated the hypothesis that these three chromatin marks define a transcription termination site in *T. brucei*. Using a panel of null mutants lacking H3v, H4v, and base J, here I show that H4v is a major sign for transcription termination at TTSs. While having a secondary function at TTSs, H3v is important for monoallelic transcription of telomeric antigen genes. The simultaneous absence of both histone variants leads to proliferation and replication defects, which are exacerbated by the J absence, accompanied by accumulation of sub-G1 population. Thus, I propose that the coordinated actions of H3v, H4v, and J provide compensatory mechanisms for each other in chromatin organization, transcription, replication, and cell-cycle progression.

## Introduction

*Trypanosoma brucei* is a parasitic protist that causes African sleeping sickness in humans and related diseases in animals, predominantly in sub-Saharan Africa. Disease transmission to mammals occurs by infected tsetse fly bites. To adapt in two different hosts, *T. brucei* undergoes stages of life cycle-specific differentiation. In the insect vector, trypanosomes proliferate as a procyclic form (PF) in the midgut and migrate to the fly’s salivary gland and differentiate into a non-proliferative metacyclic form. The infected fly injects the metacyclic form *T. brucei* into the mammalian host’s bloodstream. After differentiating into a bloodstream form (BF), trypanosomes proliferate in the host’s bloodstream and extracellular spaces. BF trypanosomes are transferred during a blood meal and differentiate into a PF and repeat the life cycle (Matthews, 2005). The surface of BF trypanosome is densely coated with a single type of variant surface glycoprotein (VSG), which can trigger a strong antibody response in the mammalian host. However, trypanosomes can escape the host’s immune system with their reservoir of over 2,500 VSG genes by sequentially expressing one VSG gene at a time, which drives disease persistence. Thus, transcriptional control of VSG genes and their genomic arrangement are key to *T. brucei* immune evasion mechanisms.

In kinetoplastid protozoa including *T. brucei*, groups of genes are assembled in polycistronic transcription units (PTUs; Johnson et al., 1987; Martìnez-Calvillo et al., 2003, 2010; Clayton, 2016, 2019). Gene transcription in *T. brucei* is controlled at three levels: (1) regulation of transcription initiation and termination at boundaries of PTUs, (2) processing of precursor RNAs (*trans*-splicing and polyadenylation) that generates mature mRNAs, and (3) regulating individual mRNA using regulatory information present in the 5′ and 3′ un-translated regions (UTRs) and *trans*-acting elements like RNA-binding proteins (Martìnez-Calvillo et al., 2010; Clayton, 2016, 2019). PTU boundaries are demarcated by specific epigenetic marks in *T. brucei*. Transcription start sites (TSSs) feature acetylated H4 (H4K10ac), methylated H3 (H3K4me3), two essential histone variants (H2Az and H2Bv), and a kinetoplastid-specific DNA modification, base J (β-D-glucosyl-hydroxymethyluracil, glucosyl-hmU; Siegel et al., 2009; Cliffe et al., 2010; Wright et al., 2010). Acetylation of H2Az is required for its deposition at TSSs. Depletion of HAT1, an acetyltransferase for H4, reduces total mRNA levels by 50% (Kraus et al., 2020). Deposition of H2Az at TSSs also requires GT-rich sequence element present in the TSSs (Wedel et al., 2017).

Transcription termination regulation in *T. brucei* remains less known. While TSSs are associated with essential chromatin factors, transcription termination sites (TTSs) feature three non-essential factors, two histone variants (H3v and H4v) and base J (Siegel et al., 2009; Cliffe et al., 2010). Base J modification uses a two-step process. J-binding protein-1 and 2 (JBP1 and JBP2) mediate thymidine hydroxylation to generate a hydroxymethyl-dU (hmU) intermediate. J-associated glucosyl transferase glucosylates hmU to generate base J (Cliffe et al., 2009; Bullard et al., 2014). Deletion of both JBP1 and JBP2 (JΔ) abolishes base J modification in *T. brucei* but allows normal growth (Cliffe et al., 2009), unlike in *Leishmania* species with essential JBP1 functions (Genest et al., 2005). Thus, base J may function differently than in the closely related kinetoplastid parasites. Approximately 50% of base J localize at boundaries of PTUs in *T. brucei*, but in *Leishmania*, only 1% of base J is chromosome internal (primarily at TTSs; van Leeuwen et al., 1997, 1998; Genest et al., 2007; Borst and Sabatini, 2008). However, this was determined before modern genomics techniques were available, and the actual percentage of chromosomal base J may be different. While J depletion significantly increased transcription readthrough at TTSs in *Leishmania* (van Luenen et al., 2012; Reynolds D. L. et al., 2016), J null trypanosomes showed minor transcription termination defects with increased antisense transcripts concentrated near TTSs (Reynolds D. et al., 2016; Schulz et al., 2016). In both organisms, no significant transcription termination defects were observed in the H3vΔ single mutant (Anderson et al., 2013; Schulz et al., 2016). However, because antisense transcript levels were higher in the H3vΔ JΔ double mutant than J null cells (Reynolds D. et al., 2016; Schulz et al., 2016), it is possible that H3v also contributes to the process of transcription termination in *T. brucei*.

The most telomere-proximal PTU in some megabase chromosomes houses a VSG, a trypanosome surface antigen gene. Allelic exclusion of VSG among the 2,500 VSG genes and periodic switching of the expressed allele allow trypanosomes to evade the host immune response, a phenomenon known as the antigenic variation. VSG genes occur at four types of chromosomal loci (Borst et al., 1983; van der Ploeg et al., 1984; Wickstead et al., 2004; Hertz-Fowler et al., 2008; Kolev et al., 2012; Cross et al., 2014): (1) bloodstream-form expression site (BES), (2) metacyclic VSG expression site (MES), (3) minichromosome (MC), and (4) subtelomeric region. The *T. brucei* genome has about 15–20 BESs, special telomeric PTUs. A BES contains a promoter associated with RNA pol I transcription (not pol II), several of expression site-associated genes, 70-bp repeats, and a VSG gene located immediately upstream of the telomere repeat. There are six MESs, each containing a VSG immediately upstream of the telomere repeat. MES VSGs are expressed specifically in the metacyclic stage by RNA pol I. *T. brucei* has about 60 small linear minichromosomes (about 30–150 kb in size). They are organized with an inverted 177-bp repeat in the middle and telomeres at the ends. Some minichromosomes have a VSG located immediately upstream of the telomere repeat, but no promoter is present (Wickstead et al., 2004). The remaining VSGs are found in arrays without promoters at subtelomeric regions. Only one BES promoter is transcriptionally active, while the others are repressed, allowing monoallelic VSG expression. A new VSG can be activated by changes in transcriptional status or genetic rearrangement that moves a silent VSG to the active VSG location (gene conversion; Machado et al., 2006). Trypanosomes coated with a new VSG are undetected by the host antibody defenses tuned to prior VSG. H3v and base J reside in telomere repeats in *T. brucei* (Siegel et al., 2009; Cliffe et al., 2010), but only the deletion of H3v caused derepression of telomeric silent VSGs (Schulz et al., 2016). The level of silent VSG derepression was higher in an H3vΔ JΔ double mutant compared to an H3vΔ single (Schulz et al., 2016), indicating that VSG silencing requires H3v with a minor contribution by J.

In contrast to TSS chromatin marks, TTS marks are non-essential (Lowell and Cross, 2004; Cliffe et al., 2009; Siegel et al., 2009; Schulz et al., 2016). I hypothesized that H3v, J, and H4v will perform redundant functions at TTSs. If this supposition were true, deleting all three marks will induce severe termination defects and result in cell lethality or sickness. I found that H3v, H4v, and base J indeed have synthetic lethal genetic interaction. H4v is a major mark of transcription termination, but H4v null cells grow normally. While having a secondary function at TTSs, H3v is important for monoallelic transcription of VSG genes. The absence of both histone variants promoted cell growth and replication defects, which increased in the absence of base J.

## Results

### Synthetic Lethal Genetic Interaction Among Transcription Termination Site-Associated Chromatin Marks

**FIGURE 1:**
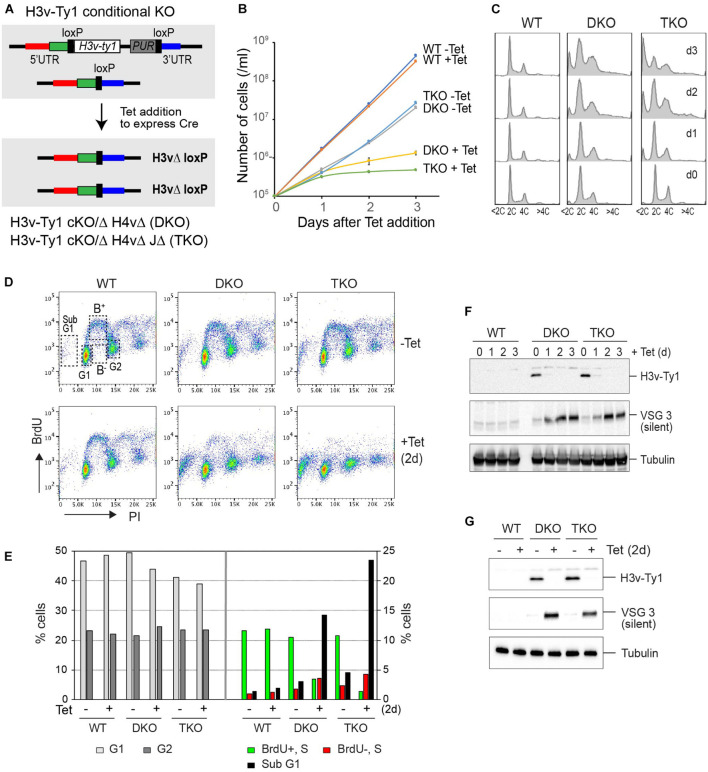
Genetic interaction between H3v, H4v, and base J in proliferation, cell-cycle progression, and DNA replication. **(A)** Strategy to generate a conditional double KO (DKO, floxed H3v-Ty1 in H4vΔ strain) and a conditional triple KO (TKO, floxed H3v-Ty1 in H4vΔ JΔ strain). The strain has one H3v allele deleted and the other H3v allele replaced with a floxed H3v-Ty1 and PUR marker. Cre protein expressed by tetracycline removes the floxed H3v-Ty1-PUR in the DKO or TKO, generating H3vΔ H4vΔ (DKO + Tet) or H3vΔ H4vΔ JΔ (TKO + Tet) cells. **(B)** Cell growth in DKO and TKO mutants upon Tet induction. WT, DKO, and TKO strains were treated with tetracycline, and cell count was monitored daily for 3 days in triplicate. **(C)** Cell-cycle progression in DKO and TKO mutants upon Tet induction. Cells from **(B)** were fixed, stained with propidium iodide (PI), and analyzed by flow cytometry. **(D)** Bromo-2′deoxyuridine (BrdU) incorporation in DKO and TKO mutants upon Tet induction. WT, DKO, and TKO strains untreated or treated with tetracycline for 2 days were pulse-labeled with 500 μM BrdU for 40 min and fixed. Cells were stained with PI (staining bulk DNA) and anti-BrdU-Alexa 488 (staining newly replicated DNA) and then analyzed by flow cytometry. **(E)** Quantification of BrdU-positive or -negative S phase cells and cells in G1, G2, or sub-G1 stage. **(F)** Western blot control for experiment B. Depletion of H3v-Ty1 protein and derepression of silent VSG3 protein in DKO and TKO mutants upon Tet induction. Denatured whole cells from **(B)** were analyzed by western blot. Tubulin served as a loading control. **(G)** Western blot control for experiment D.

Several studies have already characterized *T. brucei* mutants that were deleted for one or two genes encoding TTS mark proteins. Schulz et al. (2016) demonstrated that JΔ and H3vΔ JΔ mutants have defects in transcription termination (specifically at or near TTSs), using polyA-selected stranded RNA-seq experiments. Reynolds D. et al. (2016) also showed that J is important in transcription termination using small RNA-seq. Müller et al. (2018) showed that H3v and H4v are important for the control of VSG switching, and H3vΔ H4vΔ cells grew poorly. However, trypanosomes lacking both H3v and H4v have not been examined for transcription termination phenotypes. Although H3vΔ JΔ and JΔ mutant cells exhibited transcription termination defects, these cells grew normally. Therefore, these data suggest that successful transcription termination in *T. brucei* may require three non-essential chromatin marks (H3v, H4v, and base J) that coincide at TTSs, which may functionally “signal” RNA polymerase II to stop. Thus, the simultaneous absence of all three marks would cause severe transcription termination defects, which lead to cell growth defects. To examine whether these TTS marks have a synthetic lethal genetic interaction, I generated a full set of TTS mark knockout (KO) mutants. All experiments were performed in BF trypanosomes, as J modification is detected only in BF stage. I had previously generated H3vΔ, JΔ, and H3vΔ JΔ strains (Schulz et al., 2016), so here I generated additional KO mutants of H4vΔ and H4vΔ JΔ using an established Cre-loxP system (Kim et al., 2013a). The JΔ strain was generated by sequentially deleting both alleles of JBP1 and JBP2 genes, but this study will refer to them as a single mutant hereafter, as J is one of three TTS marks. Briefly, all parental cell lines contain a Cre recombinase gene whose expression can be induced by adding tetracycline (“Tet-on”). Gene deletion cassettes have upstream and downstream homology sequences and a floxed selection marker in the middle. After the targeted deletion of both alleles, the expressed Cre recombinase by Tet addition removed the floxed selection markers from the genome, so that they can be reused. The JΔ strain without markers served to generate H4vΔ JΔ double-mutant strains. Both H4vΔ and H4vΔ JΔ double mutants grew normally and showed no morphological abnormalities. To generate an H4vΔ H3vΔ double mutant, I transfected an H4v KO vector in the H4vΔ/+ H3vΔ strain. The six viable clones grew slower than WT and the parental strain (H4vΔ/+ H3vΔ/Δ background). I observed a clonal variation in cell growth within these clones: a group of four clones (clones 3–6) grew much slower than the other two (clones 1 and 2; Supplementary Figure 1A). To avoid any obscurity caused by unknown events occurring during prolonged period of culturing after transfection and, also, to analyze the mutant phenotypes immediately after the knockout, I generated a conditional KO mutant for H3vΔ H4vΔ double KO, a H4vΔ strain with a floxed H3v-Ty1 [conditional double KO (DKO)]. In this strain, Tet-induced Cre recombinase can remove the floxed H3v-Ty1 allele and produce H3vΔ H4vΔ cells (DKO + Tet; Figure 1A). Similarly, I attempted to generate an H3vΔ H4vΔ JΔ triple-KO strain by transfection. No viable clones emerged after deleting the remaining H4v allele in the H4vΔ/+ H3vΔ JΔ strain. So, I generated a conditional triple KO mutant, an H4vΔ JΔ strain with a floxed H3v-Ty1 [conditional triple KO (TKO)]. Genotypically, the TKO strain is a quadruple null for H3v, H4v, JBP1, and JBP2 genes.

After adding Tet, both DKO and TKO cells exhibited severe growth defects and abnormal cell-cycle distribution (greater defects in the Tet-treated TKO; Figures 1B,C). The number of cells containing less than 2C DNA content (sub-G1) significantly increased after removing the H3v-Ty1 allele in the conditional TKO strain (Figure 1C). Levels of VSG3, a silent VSG protein, increased after Tet treatment both in the DKO and TKO strains (Figure 1F). The conditional DKO double mutant grew more poorly than those generated by transfection, suggesting that cells may compensate with the loss of H3v and H4v marks in different mechanisms over time.

I observed the same sub-G1 increase in *Tb*MCM-BP-depleted cells (Kim et al., 2013b; Kim, 2019), as in the Tet-treated TKO cells. *Tb*MCM-BP depletion also caused a DNA replication defect (Kim, 2019). To determine if replication problems triggered these cell-cycle defects in TKO cells, I measured the efficiency of DNA synthesis using bromo-2′deoxyuridine (BrdU) incorporation assay. WT, DKO, and TKO cells without or with Tet treatment (2 days) were pulse-labeled with BrdU, a dT analog, which incorporates into newly replicated DNA strands. Cells were then fixed and stained with anti-BrdU antibody and Alexa 488. Bulk DNA was stained with propidium iodide (PI). Tet-untreated WT, DKO, and TKO showed a normal pattern of BrdU labeling, exhibiting the typical “horseshoe” shape in which G1 and G2 cells are BrdU-negative while most of S-phase cells are BrdU-positive. The number of BrdU-incorporating S phase cells (marked as B^+^ in Figure 1D, WT without Tet plot) was decreased in Tet-treated DKO, and this reduction was more pronounced in Tet-treated TKO (Figures 1D,E). Western blot in Figure 1G confirms the depletion of H3v-Ty1 protein. Western blot controls for cell growth curve and BrdU pulse are shown in Figures 1F,G. Levels of silent VSG3 protein increased both in the DKO and TKO strains after the depletion of H3v-Ty1 protein, indicating that VSG silencing is likely compromised in these KO cells.

### H4v Drives Transcription Termination With H3v and J Contributions

JΔ and H3vΔ JΔ mutants showed mild transcription termination defects and wild-type growth rates (Reynolds D. et al., 2016; Schulz et al., 2016). The H3vΔ H4vΔ mutant grew poorly (Müller et al., 2018), but transcription termination phenotype has not been examined. Because the Tet-treated DKO and TKO cells have growth problems (Figure 1B), I speculated that these double- and triple-KO cells would have severe transcription termination defects, which could be one of the reasons for the growth defects. To examine this possibility, I performed stranded RNA-seq experiments with triplicated cultures of WT and nine KO mutants, including JΔ, H3vΔ, H4vΔ, H3vΔ JΔ, H4vΔ JΔ, and DKO without or with Tet treatment, and TKO without or with Tet treatment. Strains were confirmed by PCR and/or western blot, flow cytometry, and also by stranded RNA-seq mapping (Supplementary Figures 2, 3). Stranded total RNA was prepared by rRNA removal to avoid the loss of antisense transcripts that are not polyadenylated or that cannot be selected with oligo dT. Sequence reads were analyzed with Bowtie 2 (Langmead and Salzberg, 2012) and SeqMonk (Babraham Bioinformatics).

**FIGURE 2:**
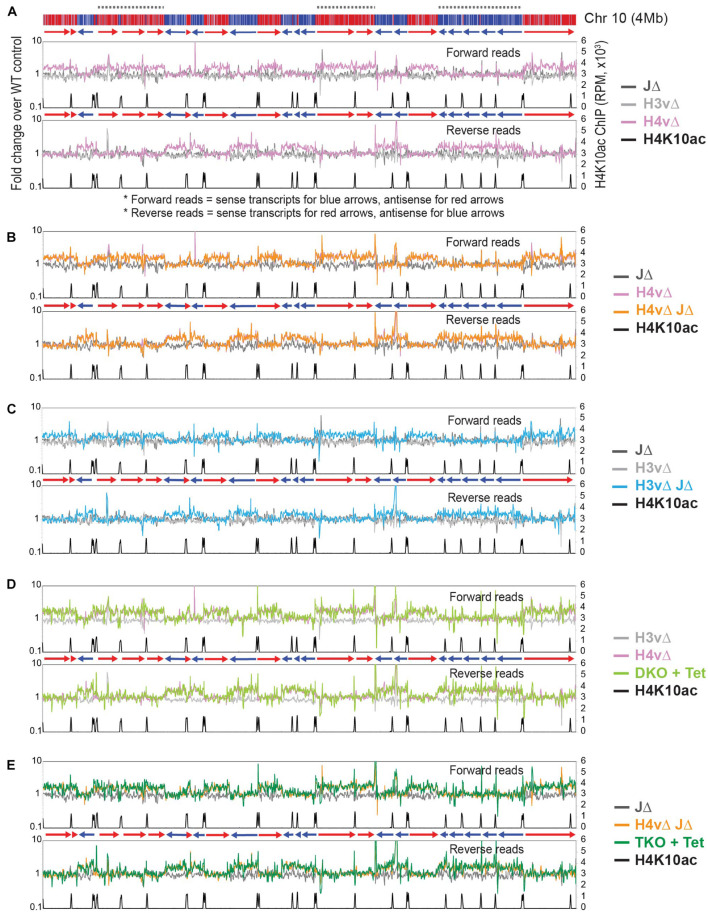
H4v is the major transcription termination signal with a secondary role of H3v and J. Global transcription was examined by rRNA-depleted stranded RNA-seq and the sequence reads mapped to the *T. brucei* Lister 427 genome. Forward and reverse reads were analyzed separately with sliding windows (5-kb bin, 1-kb step). Forward reads represent sense transcripts in reverse orientation (blue arrows, blue PTUs moving from right to left) and antisense transcripts in forward orientation (red arrows, red PTUs moving from left to right). Fold changes of reads per million mapped reads (RPM) values between WT and each of KO mutant are plotted for all chromosomes. Chromosome 10 is shown as an example. Diagram of chromosome 10 is shown on top: PTUs are shown in red and blue bars with arrow heads indicating transcription direction. Dotted lines are regions containing multiple head-to-tail (HT) PTUs in the same direction. Primary *y*-axis is a fold change of RPM values of each mutant compared to WT. Secondary *y*-axis is RPM values obtained from H4K10ac ChIP seq. H4K10ac ChIP raw reads obtained from the Cross lab (Siegel et al., 2009) were mapped to the Lister 427 genome with the Bowtie 2. The following KO mutants are compared in each plot: **(A)** JΔ, H3vΔ, and H4vΔ. **(B)** JΔ, H4vΔ, and H4vΔ JΔ. **(C)** JΔ, H3vΔ, and H3vΔ JΔ. **(D)** H3vΔ, H4vΔ, and Tet-treated DKO (H3vΔ H4vΔ). **(E)** JΔ, H4vΔ JΔ, and Tet-treated TKO (H3vΔ H4vΔ JΔ).

Stranded RNA-seq reads were aligned to the Lister 427 genome. Eleven megabase chromosomes were analyzed with sliding windows (5-kb bin; 1-kb step). Reads per million mapped reads (RPM) values were generated separately from forward and reverse reads, and fold changes compared to WT were plotted. Chromosome 10 (Figure 2) is shown as an example. TSSs are shown with H4K10ac peaks (Siegel et al., 2009). Forward reads mapping to reverse PTUs (blue; transcription moving in the reverse direction; from right to left) represent sense transcription, and forward reads mapping to forward PTUs (red; transcription moving from left to right) represent antisense transcription. Reverse reads mapping to forward PTUs (red) represent sense and to reverse PTUs represent antisense transcription. Transcript levels were unchanged in JΔ and H3vΔ, compared to WT (Figure 2A). H4vΔ (pink lines) showed an approximately two-fold increase in antisense transcript levels throughout the entire chromosome 10. In the region for PTU assembly in head-to-tail organization (HT; two PTUs moving in the same direction so the HT sites have TTS and TSS chromatin marks next to each other; Figure 2; dotted line in chromosome 10 diagram), all PTUs have the same increase in antisense transcript levels. This indicates that a readthrough transcription does not stop when it reaches the TTS of the second head-to-tail PTU (where H4v is absent) and continues to travel until it reaches the first divergent TSS. If readthrough machineries terminate at random positions, levels of antisense transcript increase should fluctuate. If they continue to the ends of chromosome, levels of antisense transcripts should be higher at PTUs located near the ends of chromosome. Thus, the data suggests that H4v is an important signal for transcription stop and that the readthrough transcription machinery traveling in the wrong direction may fall off at the first divergent TSS that it meets. It could be because RNA pol II directionality is determined at divergent TSSs (Marquardt et al., 2014; Bagchi and Iyer, 2016; Wedel et al., 2017).

No further increase in antisense transcription was found in H4vΔ JΔ compared to H4vΔ strain (Figure 2B). The same pattern of antisense-transcription increase occurred in H3vΔ JΔ strain (Figure 2C), but to a lesser degree. Because H3vΔ or JΔ alone did not show significant changes in antisense transcription, one possibility is that all three marks independently control transcription termination. This predicts that levels of antisense transcripts should be higher in the Tet-treated DKO or TKO than in H4vΔ or H4vΔ JΔ mutant (additive or synergistic effect). The second possibility is that H4v is the major signal for termination with assistance from H3v and base J. If so, H4vΔ H3vΔ and H4vΔ H3vΔ JΔ mutants should show similar levels of antisense transcripts as the H4vΔ. As shown in Figures 2D,E, the increase in antisense transcription was comparable between H3vΔ H4vΔ (DKO + Tet) and H4vΔ and between H3vΔ H4vΔ JΔ (TKO + Tet) and H4vΔ JΔ. The same trend was observed in all chromosomes (plots are not shown). Therefore, H4v is the major chromatin mark for transcription termination with secondary contributions from H3v and J. The transcription termination problem is not the only cause for the cell growth defects in the DKO and TKO cells.

Reads per kilobase per million mapped reads (RPKM) values of all, sense, or antisense reads mapping to 8,428 coding sequences (CDSs; excluding those located in subtelomeric regions) are presented in Supplementary Figure 5A. Mean values of antisense transcription increased about 1.44-fold (H3vΔ JΔ), 1.64-fold (H4vΔ), 1.75-fold (DKO + Tet), and 1.77-fold (TKO + Tet) compared to WT, while sense transcription did not significantly change. The differences are statistically significant (Supplementary Table 7). *P* values were generated from Kruskal–Wallis one-way analysis of variance test across all genotypes and from Mann–Whitney *U* test comparing mutants to WT for CDS levels. Location of CDSs with up-regulated antisense transcription confirmed that the increases in antisense transcripts are global and evenly distributed throughout all PTUs (Supplementary Figure 5B). It is possible that the actual increase in transcript levels could be even higher, because RNA stability also affects the level of transcripts measured in RNA-seq.

### Transcription Termination Site Chromatin Marks Affect Levels of polyA-Selected Antisense Transcripts

**FIGURE 3:**
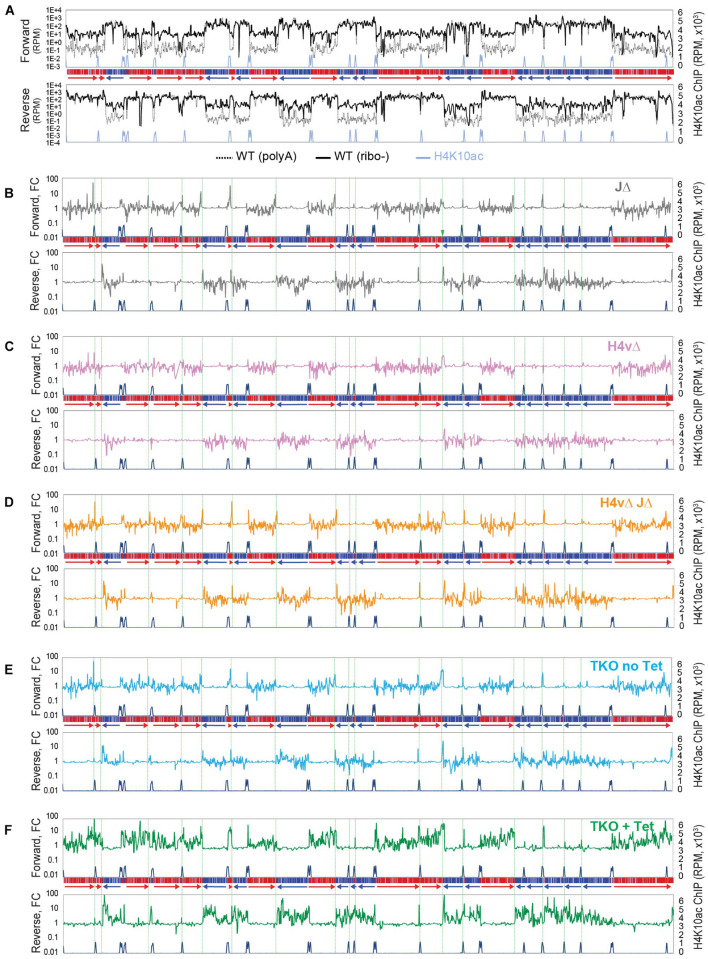
Transcription termination site (TTS) chromatin marks affect levels of polyA-selected antisense transcripts. Global transcription was examined by polyA-selected stranded RNA-seq and mapped to the Lister 427 genome. **(A)** Forward and reverse reads were analyzed separately with sliding windows (5-kb bin, 1-kb step). RPM values from WT RNA-seq prepared by polyA selection (dotted black line) were compared with those from WT rRNA-depleted RNA-seq (solid black line). RPM values were plotted for all chromosomes. Chromosome 10 is shown as an example. A diagram of chromosome 10 is depicted in the middle of forward and reverse plots. **(B–F)** Fold changes between wild-type and each KO mutant are plotted. The shown chromosome 10 are as follows: JΔ **(B)**, H4vΔ **(C)**, H4vΔ JΔ **(D)**, TKO without Tet **(E)**, and TKO with Tet **(F)**. Green dotted lines indicate locations of TTSs.

We demonstrated that antisense transcription was significantly increased at and near TTSs in J null cells using polyA-selected stranded RNA-seq experiments (Schulz et al., 2016). In the rRNA depletion method here, antisense transcription showed minimal alterations in JΔ mutant cells (Figure 2). This suggests that TTS chromatin marks may be involved also in polyadenylation of antisense RNA. So, I first compared WT transcription profiles obtained from polyA-selected and rRNA-depleted stranded RNA-seq experiments. Reads mapped to the Lister 427 genome were analyzed with sliding windows (5-kb bin; 1-kb step), as in Figure 2. RPM values for each method were plotted for chromosome 10 (Figure 3A). Forward read mapping represents sense transcription for reverse PTUs (blue) and antisense for forward PTUs (red), and vice versa for reverse read mapping. For sense transcription, I found no change between samples prepared by polyA selection and rRNA depletion, but a higher level of antisense transcripts (about 10–100-fold) was detected in rRNA-depleted samples compared to polyA-selected samples. Thus, this result indicates that our previous method (Schulz et al., 2016) was able to detect only a fraction of antisense transcripts. Because not much is known for mechanisms of polyadenylation of antisense transcripts in *T. brucei*, we do not know whether antisense transcripts detected from this seq method are products of polyadenylation and *trans*-splicing as the sense transcripts.

Since JΔ increased antisense transcripts near termination sites in the polyA selection method, while JΔ did not exhibit significant changes in antisense transcript levels in rRNA-depletion method (Figure 2), it is possible that the polyA selection method could allow to detect different phenotypes in the H4v KO mutants. Thus, I analyzed some of H4vΔ mutants in polyA-selected stranded RNA-seq experiments. Levels of polyA-selected antisense transcripts increased near TTSs in J Δ (spiking peaks near TTSs: green lines in Figure 3B), consistent with prior results (Schulz et al., 2016). This was also observed in H4vΔ JΔ and TKO without Tet (Flag-H4v cKO in H3vΔ JΔ; Figures 3D,E). Interestingly, Tet-treated TKO cells exhibited a substantial increase in the levels of polyA-selected antisense transcripts, and these increases were not only localized at TTSs but also distributed to all PTU regions (Figure 3F). H4vΔ alone did not significantly change the level of polyA-selected antisense transcripts (Figure 3C). The stranded RNA-seq data (Figures 2, 3) indicate that H4v serves as a major mark for transcription termination, but all TTS marks may independently contribute to the post-transcription regulation of antisense transcripts, and they have more functions in transcription control that remain to be identified. Further studies are also necessary to determine whether this increased level of polyA-selected antisense transcripts contributes to the cell growth defects in the triple KO.

A transcriptome analysis of 8,428 CDSs showed that antisense transcription was 1.69-fold higher in the triple-KO mutant compared to WT (Supplementary Figure 5C). Statistical analyses for all transcriptome data are summarized in Supplementary Table 7. The differences are statistically significant. *P* values were generated from Kruskal–Wallis one-way analysis of variance test across all genotypes and from Mann–Whitney *U* test comparing mutants to WT for CDS levels.

Levels of antisense transcripts increased in the Tet-treated TKO cells in both RNA-seq experiments. One would expect that increased levels of antisense RNA could disrupt the transcriptome *via* RNAi interference by formation of dsRNA. However, levels of sense transcripts were not significantly changed in the Tet-treated TKO in both RNA-seq experiments, suggesting that increased antisense transcription does not substantially interfere with sense transcript stability.

### Transcription Termination Site Chromatin Marks Control Variant Surface Glycoprotein Transcription in a Synergistic Manner

**FIGURE 4:**
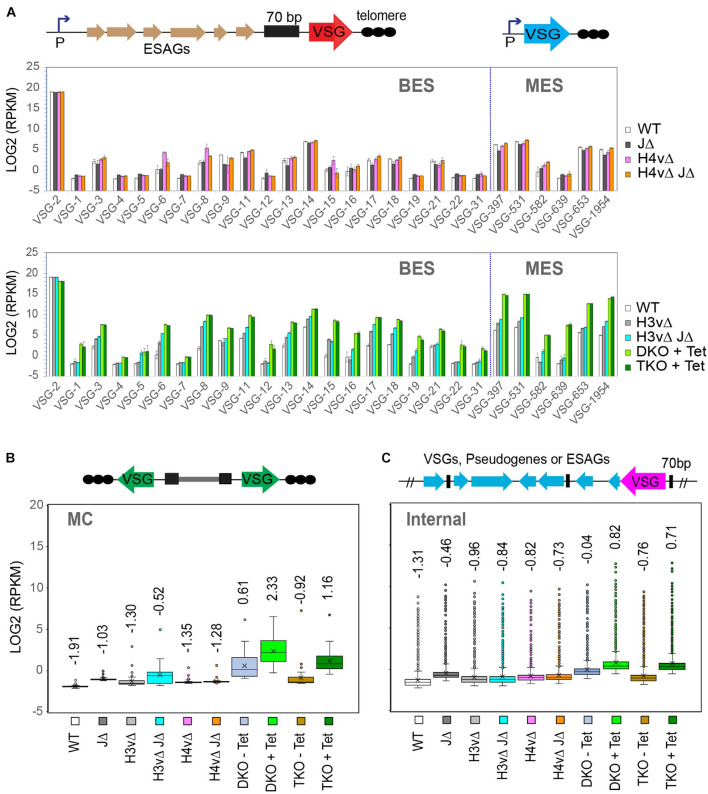
TTS chromatin marks are requisite for variant surface glycoprotein (VSG) transcription in a synergistic manner. VSG RNA levels in WT and KO mutants were analyzed by rRNA-depleted stranded RNA-seq. RNA-seq reads were aligned to the VSGnome database (Cross et al., 2014), and LOG_2_(RPKM) values were obtained for each VSG in wild-type and KO mutants. **(A)** Bar graphs comparing WT and KO mutants for bloodstream-form expression site (BES) and metacyclic VSG expression site (MES) VSG expression levels. Mutants are grouped by the loss of VSG silencing phenotype: mutants that showed no change (top; including JΔ, H4vΔ, and H4vΔ JΔ) and mutants that increased the expression of silent VSGs (bottom; including H3vΔ, H3vΔ JΔ, DKO + Tet, and TKO + Tet). Error bars indicate standard deviation between three replicates. Diagrams of BES and MES are shown. **(B)** Box plot comparing WT and KO mutants for minichromosomal (MC) VSG expression levels. **(C)** Box plot comparing WT and KO mutants for chromosome internal VSG expression levels. Statistical analyses are summarized in Supplementary Table 7.

While chromosome internal TTSs have all three marks, telomeres are enriched with H3v and base J, but not H4v (Lowell and Cross, 2004; Siegel et al., 2009; Cliffe et al., 2010). Transcription is repressed at telomeric regions by a heterochromatin structure in *T. brucei* as in other eukaryotes (Ottaviani et al., 2008; Cestari and Stuart, 2018; Janssen et al., 2018), and transcription of telomeric VSG utilizes RNA pol I (Gunzl et al., 2003), not pol II. Therefore, I speculated that VSG transcription would be controlled differently from the transcription of chromosome internal PTUs driven by RNA pol II. Four types of VSG locations are depicted in Figure 4. A single VSG is transcribed from one of the 15–20 BESs (telomeric PTUs with pol I promoter 10–60 kb in length; Gunzl et al., 2003; Hertz-Fowler et al., 2008; Horn and McCulloch, 2010; Cross et al., 2014). Additional genomic locations that contain VSGs are also telomeric [MES (Kolev et al., 2012; Cross et al., 2014) or MCs (Wickstead et al., 2004)] or subtelomeric [chromosome internal VSGs (mostly partial genes or pseudogenes)]. Only one BES is transcriptionally active at a time, while the remaining BESs are silent.

RNA-seq reads were mapped to the VSGnome (Cross et al., 2014), and LOG_2_(RPKM) values of VSG CDSs were compared between WT and KO mutants for BES and MES VSGs (Figure 4A). H4vΔ or H4vΔ JΔ cells exhibited no significant changes in levels of silent BES and MES VSG transcripts (the graph in the top panel of Figure 4A). Mutants lacking H3v showed increased levels of silent BES and MES VSGs, in the order of DKO + Tet, TKO + Tet > H3vΔ JΔ > H3vΔ (high to low; the graph in the bottom panel of Figure 4A). Given that H4vΔ and H4vΔ JΔ showed no change in VSG silencing and H4v does not localize at the telomere, the synergistic upregulation of silent ES VSGs in Tet-treated DKO and TKO cells was puzzling. It is possible that H4v could bind telomere but are not detectable or that H4v may bind telomeres only in the absence of H3v.

Although minichromosomal VSGs do not have a promoter, they experience a similar regulation by H3v, H4v, and J (Figure 4B). The mean values of minichromosomal VSG transcript levels increased in mutants lacking H3v, in the order of DKO + Tet, TKO + Tet, H3vΔ JΔ, and H3vΔ (high to low), though the underlying mechanism for the derepression of these promoter-less VSGs remains unclear. They may contain “promoter-like” elements, which become more accessible by RNA polymerases, as the heterochromatin structure is disrupted in H3vΔ H4vΔ cells. It is known that the chromatin opening can result in RNA pol II transcription initiation (McAndrew et al., 1998). Chromosome internal VSGs were also up-regulated similarly (Figure 4C). Statistical analyses for all VSG expression data are in Supplementary Table 7.

The percentage of each VSG type, the active VSG2, silent BESs, MESs, minichromosomal, and internal VSGs is plotted in Supplementary Figure 6A. In WT, 99% of VSG transcripts were from the active VSG2, but in Tet-treated DKO and TKO cells, only 67–68% was the active VSG2 and ∼30% was from silent VSGs. About 20% of silent VSG transcripts were from MES VSGs in the Tet-treated DKO and TKO, indicating that H3v, H4v, and J have major roles in the MES silencing control. Compared to WT, MES VSGs were 312- and 307-fold up-regulated, and BES VSGs were 36- and 33-fold up-regulated in the Tet-treated DKO and TKO strains, respectively (Supplementary Figure 6B). Transcription at the entire BES units was also examined. Interestingly, only those genes adjacent to promoter or telomere (VSG) were significantly affected by H3v, H4v, and J (Supplementary Figure 6C). A possible explanation would be that in the absence of H3v and H4v, telomere repeats are devoid of nucleosomes, which may disrupt heterochromatin structure of silent ESs and lead to the loss of VSG and promoter silencing.

Müller et al. (2018) observed that H3vΔ H4vΔ cells switch at a higher rate than WT. To determine if VSG switching also increases in Tet-treated DKO and TKO cells, I examined the rate of VSG switching using flow cytometry. Because removing H3v is not reversible and Tet-treated conditional KO cells are very sick, accurately measuring the VSG switching rate posed challenges. Therefore, I looked at switching between two VSGs (from VSG2 to VSG3) by staining them with antibodies conjugated with different fluorophores, the active VSG2 with Dylight 488 (green) and the silent VSG3 with Dylight 650 (red). VSG3 was chosen because it is the most frequently selected silent VSGs in switching assays in the single marker (SM) strain background (Kim and Cross, 2010; Hovel-Miner et al., 2012, 2016; Schulz et al., 2016). VSG3-expressing cells were added into VSG2-expressing cells at 0.01, 0.1, 1, and 10%, and cells were stained with anti-VSG2 and anti-VSG3 antibodies and analyzed by flow cytometry. The percentage of mixed-in VSG3-expressing cells matched well with the percentage of VSG3 detected (Supplementary Figures 7A,B). However, mixing of two strains expressing two different VSG coats produced a population of cells that colocalized both antibodies. This occurred only when two strains were mixed, as they were not detected in 100% VSG2-expressing cells (VSG2 only). Using this method, silent VSG3 expression (derepression or switching) was examined in WT, DKO, and TKO treated with Tet for 2 days or without Tet (Supplementary Figure 7C). The number of double-positive cells increased in Tet-treated DKO and TKO cells, but I did not detect any obvious VSG3 switchers in either sample. At least for VSG3, switching did not substantially increase. However, some population of Tet-treated DKO and TKO cells exhibited an intermediate level of VSG2 staining. Because the half-life of VSG2 protein is 4.5 h with a normal growth rate (Pinger et al., 2017), depending on when the switching event occurred, some of switched cells could still have the old VSG proteins on the surface. Thus, it is possible that cells with an intermediate level of VSG2 signal might be switchers. Since the metacyclic VSG transcripts increased about 300-fold in the Tet-treated DKO and TKO mutants (Supplementary Figure 6B), antibodies against metacyclic VSGs would produce more interpretable data (but such tools are unavailable now). Alternatively, these could be abnormal cells that are auto-fluorescent. More studies will be necessary to determine the VSG switching rate in the triple-KO mutant.

### Global DNA Replication Impairment in Triple Knockout Cells

The triple-KO cells showed inefficient DNA replication and abnormal cell division, producing a large number of cells containing <2C DNA content. *Tb*ORC1 associates with most of TSSs (both divergent TSSs and HT sites) and a few of TTSs. About 40 (∼25%) of ORC1-binding sites are early replicating origins (Tiengwe et al., 2012). Therefore, transcription must coordinate with replication progression to prevent genome instability. To investigate global replication initiation at early replicating origins, I performed a MFA-seq experiment (marker frequency analysis followed by high-throughput sequencing) in TKO cells, as previously described (Tiengwe et al., 2012; Devlin et al., 2016; Kim, 2019).

**FIGURE 5:**
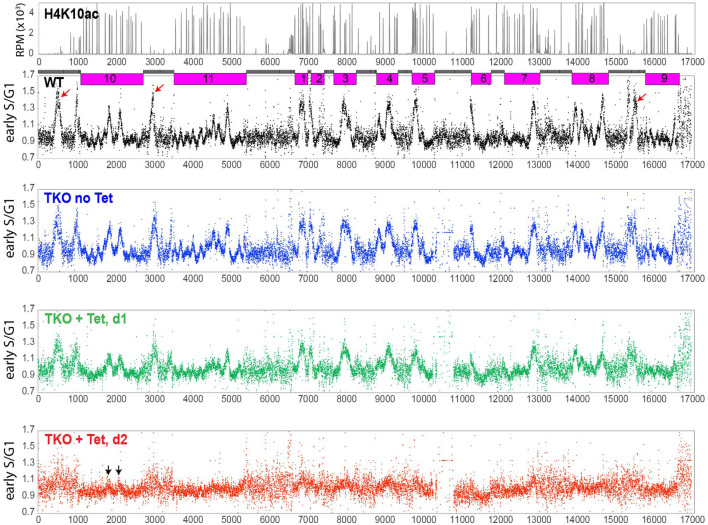
Global DNA replication impairment in TKO cells. Replication was examined by marker frequency analysis followed by high-throughput sequencing (MFA-seq). TKO strain with a floxed H3v-HA allele was treated with tetracycline for 0, 1, or 2 days and stained with PI. Cells in G1, early S, late S, and G2 phase were FACS-sorted (Supplementary Figure 8C). Genomic DNA was prepared and sequenced in the Illumina platform. Sequence reads were aligned to the Lister 427 genome and analyzed with sliding window (10-kb bin, 2.5-kb step). Read count ratio between early S to G1 was plotted for the whole genome, including subtelomeric regions. Chromosome cores (pink) and subtelomeres (gray) in the chromosome diagram are shown. Several of early replicating origins occur in subtelomeres of chromosomes 10, 11, and 9 (red arrows in the WT plot). MFA-seq reads obtained from WT (Kim, 2019) were re-analyzed and mapped to the Lister 427 genome for comparison. Subtelomere 6A of chromosome 6 has been lost in the TKO strain.

Propidium iodide-stained TKO cells treated with tetracycline for 0, 1, and 2 days were sorted by fluorescence-activated cell sorting (FACS; Supplementary Figure 8). Genomic DNA was isolated from cells in G1, early S, late S, or G2 phase and sequenced. Sequence reads were mapped to the Lister 427 reference genome and analyzed with sliding windows (10-kb bin; 2.5-kb step; Figure 5). I re-analyzed WT MFA-seq reads [which were previously mapped to the *Tb*927v5 reference genome (Kim, 2019)] for the Lister 427 genome. I found that subtelomeres also contained strong early firing origins (indicated as arrows in red in WT). Untreated TKO strain showed the same peak pattern as the WT. At day 1 post Tet induction, the peak pattern was the same but with a reduced intensity compared to day 0. Although several of small peaks were visible at some of strong origin sites at day 2 (indicated as arrows in black), no major peaks were detected, indicating a global impairment of DNA replication initiation.

Cell-cycle sorting (Supplementary Figures 8C,D) indicates that early-S phase cells should yield about 30% more DNA compared to G1 cells. While this increased DNA amount was clearly mapped to regions of early replicating origins in WT and untreated TKO cells, in TKO cells treated with Tet for 2 days, it is impossible to identify regions where this extra DNA was generated from, as no peak was detected. One possibility is that DNA synthesis may be initiated promiscuously (e.g., activating more origins including dormant ones), but replication forks progress inefficiently, producing shorter BrdU-labeled strands. Alternatively, unequal chromosome segregation in a replication-defective parent cell may produce daughter cells with one containing more DNA (early-S DNA content) and the other containing less DNA (sub-G1 DNA content).

The largest subunit of replication protein A complex (RPA), RPA1, forms nuclear foci during replication stress and DNA damage in eukaryotes, including *T. brucei* (Broderick et al., 2010; Allen et al., 2011; Marin et al., 2018). Tet-treated TKO cells displayed nuclear *Tb*RPA1 foci (Supplementary Figure 8D). Collectively, the data suggest that the loss of TTS marks induces replication stress and activates the DNA damage response (DDR) including *Tb*RPA1 recruitment to the lesions.

### Emergence of Survivors After the Removal of H3v in Double Knockout and Triple Knockout Mutants

**FIGURE 6:**
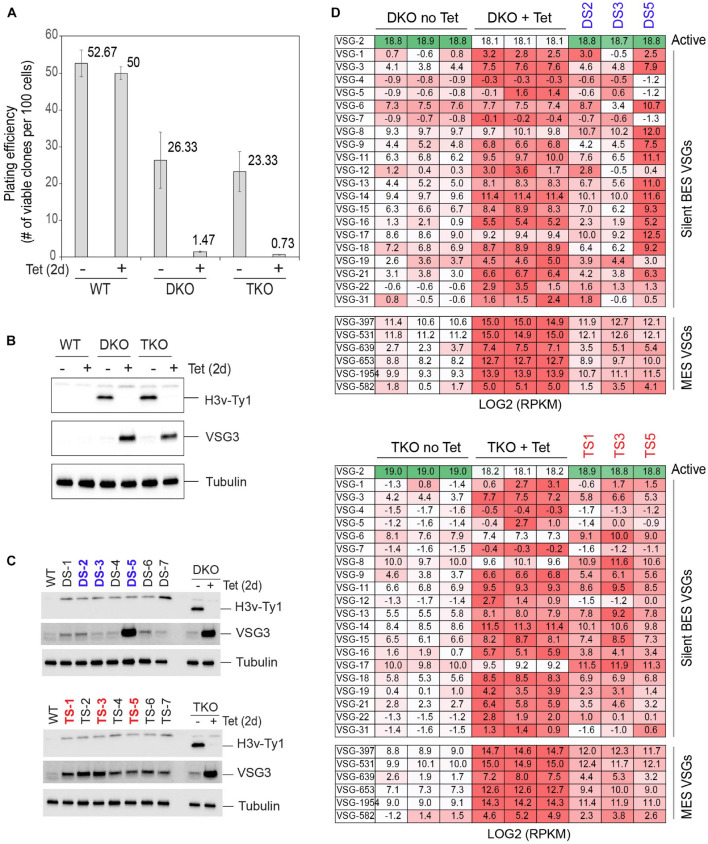
Emergence of survivors after the removal of H3v in DKO and TKO mutants: VSG silencing phenotypes. **(A)** Plating efficiency after H3v-Ty1 removal in DKO and TKO strains. WT, DKO, and TKO strains were treated with tetracycline for 2 days to remove H3v-Ty1 allele. One hundred or 1,000 cells were distributed in 96-well plates, and the number of wells containing live cells was counted to determine the viability. Clones that are resistant to puromycin were excluded as they may grow because they still retain the H3v-Ty1-PUR allele. Error bars indicate standard deviation between three replicates. **(B)** Western blot confirming the absence of H3v-Ty1 protein in Tet-treated cells in **(A)**. **(C)** Silent VSG3 protein expression in survivors emerged after removing H3v-Ty1. Seven of survivor clones from DKO (DS 1–7) or TKO (TS 1–7) were examined for silent VSG3 expression by western blot. **(D)** VSG silencing in DS and TS clones was examined by stranded RNA-seq (rRNA depletion). Heat maps compare DKO replicates (-/+ Tet) and DS clones and TKO replicates (-/+ Tet) and TS clones for BES and MES VSG transcript levels (darker color = higher expression level).

H3vΔ H4vΔ cells generated by conditional KO showed defects in growth, cell cycle, and DNA replication within 2 days (Figure 1). Growth stalling occurred in some of H3vΔ H4vΔ clones obtained by transfection (Supplementary Figure 1). These data suggest that simultaneous removal of H3v and H4v may cause growth arrest initially, but some cells adapt and manage to proliferate. To determine whether Tet-treated DKO cells are inviable or growth arrested, I measured the viability of DKO and TKO cells 2 days after Tet induction. The viability of Tet-treated DKO and TKO mutants was both low (Figures 6A,B), indicating that H3vΔ H4vΔ is lethal primarily (94.4 and 96.9% lethal). Seven survived clones from each strain (DKO survivor, DS and TKO survivor, and TS) were analyzed. They grew at different rates (Supplementary Figure 9A). DS clones showed different levels of VSG3 proteins, while TS clones showed similar levels of VSG3 protein but lower than the Tet-treated TKO control (Figure 6C). The level of VSG3 protein was higher in the DS5 compared to the rest of the DS clones. Thus, the DS5 and two DS clones in random and three TS clones in random were selected for further analysis with stranded RNA-seq (rRNA depletion). DS clones showed different patterns of BES VSG expression (Figure 6D, top). In DS2 and DS3, silent VSG expression levels were lower than Tet-treated DKO replicates, but majority of silent VSGs in DS5 expressed the same level or more than that in Tet-treated DKO cells. The levels of MES VSG transcripts in DS clones were lower than in Tet-treated DKO replicates and were generally uniform. Compared to DS clones, less clonal variation occurred in TS clones in BES or MES VSG expression (Figure 6D, bottom). VSG expression profiles of all replicates are summarized in box plots (Supplementary Figure 10).

**FIGURE 7:**
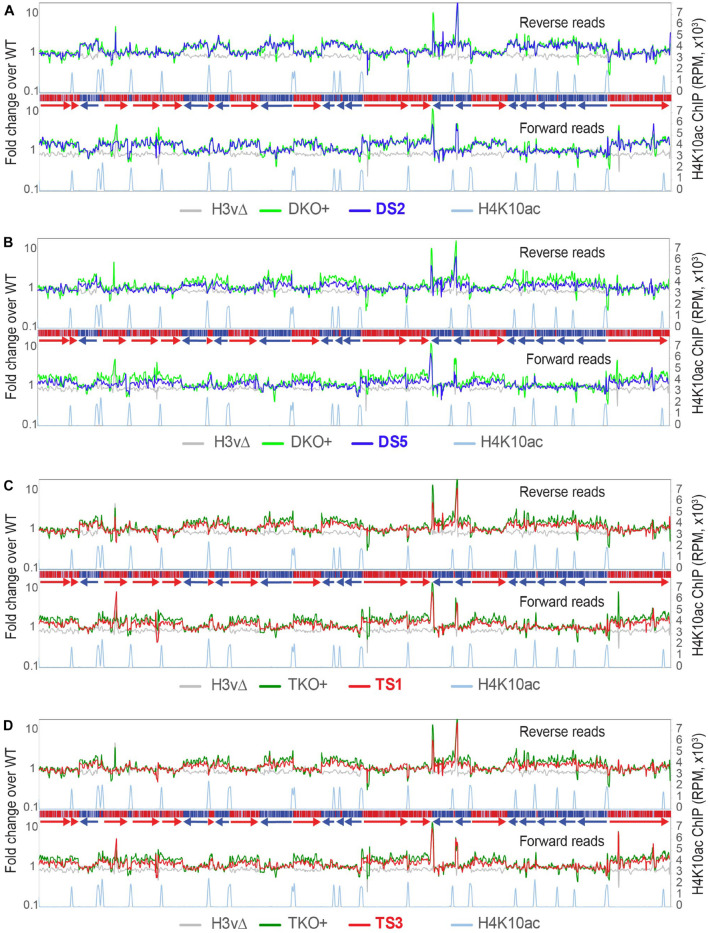
Emergence of survivors after the removal of H3v in DKO and TKO mutants: transcription phenotypes. Global transcription and antisense transcription were examined in three DS and three TS clones by rRNA-depleted stranded RNA-seq (10-kb bin, 2.5-kb step). Fold changes compared to WT were plotted and compared with Tet-treated DKO or TKO mutant. Two DS (DS2 and DS5) and two TS (TS1 and TS3) clones are shown.

Because H4v is the main factor for transcription termination control, I did not suspect major changes in transcription profiles, as H4v was already deleted in these DKO and TKO strains. Interestingly, transcription termination defects were partially alleviated in the DS5 clone, while no major change was observed in DS2 (Figures 7A,B). The levels of elevated antisense transcripts in TS clones were slightly lower than in Tet-treated TKO throughout the genome (Figures 7C,D, chromosome 10 shown).

### Complementation of Triple Knockout Phenotypes by H3v

**FIGURE 8:**
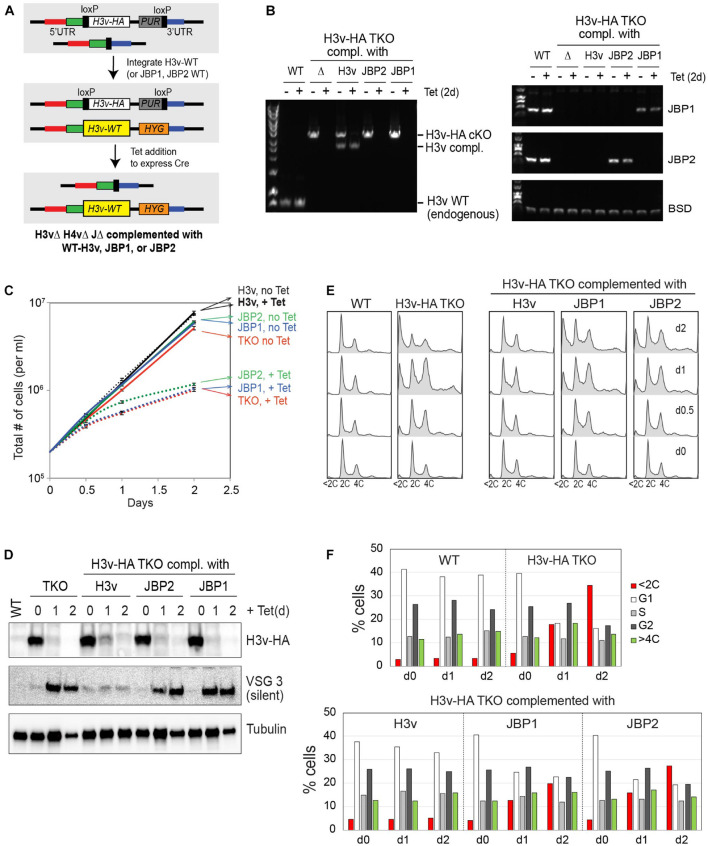
Full and partial complementation of TKO phenotypes by H3v and JBP1/2. **(A)** A strategy to generate complemented cell lines. WT-H3v, JBP1, or JBP2 gene was reintroduced to the H3v-HA TKO strain (floxed H3v-HA in H4vΔ JΔ strain) at the endogenous H3v locus where the H3v was deleted and the selection marker removed. Because the targeting vectors do not have loxP sites, Cre cannot remove the complementing gene, while it removes floxed H3v-HA allele. Resulting cells will express WT-H3v, JBP1, or JBP2 in H3vΔ H4vΔ JΔ background. **(B)** PCR genotyping confirming the removal of the floxed H3v-HA allele and the presence of complemented H3v, JBP1, or JBP2 gene in Tet-untreated or Tet-treated TKO strains. **(C)** Complementation of growth defect. TKO or TKO strain integrated with WT-H3v, JBP1, or JBP2 was treated with tetracycline for 0, 0.5, 1, and 2 days as the cell count was monitored. **(D)** Complementation of silent VSG3 derepression. H3v-HA and VSG3 protein expression by western blot. **(E)** Complementation of cell-cycle defect. Cells treated as in **(C)** were fixed and stained with PI and analyzed by flow cytometry. **(F)** Graphs showing percentage of G1, G2, S, and cells with abnormal DNA content (e.g., <2C or >4C).

To ensure that the phenotypes observed in the Tet-induced TKO strain were due to the H3v removal and not some artifacts, I conducted complementation experiments. I re-introduced an “un-floxed” WT-H3v allele in the TKO strain (floxed H3v-HA/Δ H4vΔ JΔ; Figure 8A). The induced Cre recobminase can remove only the floxed H3v-HA allele and not the complementing WT-H3v, as shown in PCR genotyping (Figure 8B). H3v fully complemented the triple KO’s defects in cell growth, cell-cycle progression, and VSG silencing (Figures 8C–F), indicating that H3v removal caused the observed phenotypes. WT-JBP1 or JBP2 gene was also re-introduced in the TKO strain using the same approach. Slight growth improvements were observed in the JBP2-complemented TKO cells. JBP2 partially rescued the cell-cycle defect (Figures 8C–F), as the number of sub-G1 cells was significantly reduced by JBP2 introduction. JBP1 also reduced the number of sub-G1 cells, but fewer than JBP2. Neither JBP1 nor JBP2 could rescue the VSG silencing defect of the TKO (Figure 8D). Re-introduction of JBP1 or JBP2 restored the J modification in the J null cells at different genomic loci (Cliffe et al., 2009, 2010). JBP1 stimulated base J modification at PTU boundaries, including TTSs and TSSs, while JBP2 did not (Cliffe et al., 2010). Instead, JBP2 partially restored J modifications associated at telomere, 70-bp repeats, and 177-bp repeats (Cliffe et al., 2009). The data suggests that a global restoration of J modification both at PTU borders and telomere may be required for the full complementation of the TKO.

## Discussion

The data here indicate that the coordinated actions from TTS chromatin marks are required for the regulation of transcription, replication, and cell-cycle progression. A global increase in the antisense transcript levels was observed in the absence of H4v in rRNA-depleted stranded RNA-seq, but no significant change was observed in the H4vΔ by polyA-selected stranded RNA-seq. Interestingly, while the triple KO showed a similar level of increase in antisense transcription as the H4vΔ with the rRNA depletion method, it showed a substantial increase in antisense transcripts by polyA selection method. The data suggest that H4v is the major signal to stop transcription termination at TTSs, and TTS marks may involve in RNA processing events occurring after transcription.

TFIIS2-2 (transcription elongation factor) is enriched at TTSs and interacts with PAF1 complex in *T. brucei* (Staneva et al., 2021). PAF1 contributes to chromatin remodeling, transcription elongation, and polyadenylation in yeast and mouse cells (Penheiter et al., 2005; Yang et al., 2016). Interestingly, PAF1 complex interacts with JBP3 in *Leishmania tarentolae* (Jensen et al., 2021), and JBP3 depletion in *L. tarentolae* and *T. brucei* led to transcription readthrough at TTSs (Kieft et al., 2020; Jensen et al., 2021). In plants, polyadenylation of antisense transcripts affects sense transcript level by interacting with RNA-binding protein, RNA 3′ processing factor, histone H3 methyltransferase, and demethylase (Fang et al., 2020). Further studies are necessary to understand why more polyA-selected antisense transcripts are produced in the absence of TTS marks and whether these polyA-selected antisense transcripts in the triple KO modulate cell viability and also to determine underlying mechanisms of antisense transcript processing.

**FIGURE 9:**
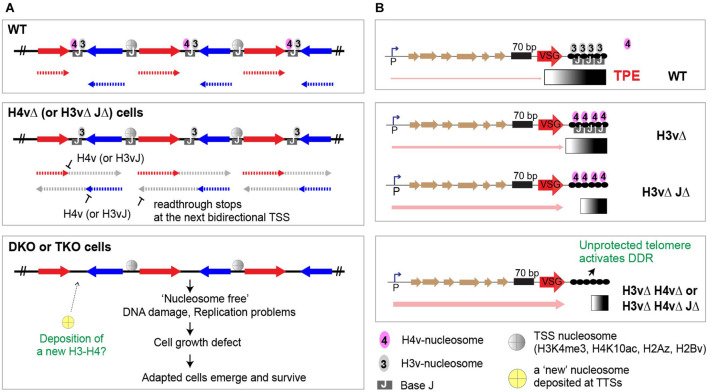
Roles of H3v, H4v, and base J in transcription and genome maintenance. **(A)** At TTSs, J-modified DNA wraps around nucleosomes containing H3v or H4v. H4v nucleosomes signal the RNA pol II machinery to stop at TTSs. Pol II that missed the stop sign continues to progress until it reaches the next divergent transcription start site (TSS). Simultaneous absence of H3v and J affects H4v nucleosome function by changing the TTS chromatin structure. Upon removal of H3v in DKO or TKO cells, nucleosome-free TTS DNA becomes fragile, accumulates replication stress and DNA lesions, and activates DNA damage response (DDR). A cell may deposit a functional pair of H3-H4 PTMs. A survivor clone with new epigenetic marks emerges if it could form stable nucleosomes and TTS chromatin structure and inherit. **(B)** The synergistic effect caused by H3v and H4v double KO in VSG silencing suggests that H4v nucleosome may bind telomere repeats in the absence of H3v. The loss of both H3v and H4v nucleosomes at telomeric regions could leave telomeres unprotected, leading to chromosome abnormalities, disruption of heterochromatin structure, and loss of VSG silencing. Differing degrees of VSG derepression level between BES VSGs in a DS clone and also between DS clones suggest that individual telomere in survivors may not have the same epigenetic code.

H3v and H4v deletion led to problems in DNA synthesis and growth in the WT and J null background. However, about 5.6 and 3.1% of Tet-treated DKO and TKO cells produced viable clones, respectively. Since the high rate suggested another mechanism than genetic suppression, I hypothesized that epigenetic adaptation may underlie this effect. The initial outcome of H3v removal in the DKO and TKO strains could leave TTS regions unprotected by nucleosomes, which can be detrimental to the cells. In mice, cells with less nucleosomes accumulated more DNA damage, and nucleosome-occupied regions had more protection from irradiation-induced DNA damage (Brambilla et al., 2020). Therefore, nucleosome-free TTS DNA may become fragile, accumulate DNA lesions, and activate DDR in Tet-treated DKO and TKO cells. To protect TTS DNA, cells may attempt to randomly deposit core H3-H4, perhaps with specific PTMs at TTSs, most of which are likely unsuccessful. If a cell deposited a functional pair of H3-H4 PTMs capable of stable nucleosome formation at TTSs, these cells could emerge and survive (Figure 9A). Several H3v and H4v PTMs are now identified (Kraus et al., 2020), although their functions are not yet known, as well as chromatin proteins associated at TTSs, which include readers and writers of histone PTMs (BDF7, PHD2, PHD4, and DOT1A; Staneva et al., 2021). H4 (or H3) with a specific PTM may associate with H3v (or H4v) for depositing to TTSs. PTMs of histones and histone variants marking TTSs may play critical roles for regulating transcription termination.

Sub-G1 population increase was more pronounced in Tet-treated TKO, than DKO, indicating that J may have roles in cell-cycle control. In other eukaryotes, activation of cell-cycle checkpoint upon DNA damage or replication stress leads to cell-cycle arrest at G1 or G2/M, or delays in S-phase progression (Waterman et al., 2020). Thus, the accumulation of sub-G1 cells in this *T. brucei* mutant is an unusual phenotype for a checkpoint-defective mutant. Sub-G1 cells could arise through an unequal segregation of chromosome and/or uncoupling of mitosis/cytokinesis and replication completion. Alternatively, the sub-G1 cells could arise as a secondary effect of cell sickness and/or death. Whether JBP proteins have direct roles in cell-cycle control in the absence of H3v and H4v remains to be elucidated.

*Trypanosoma brucei* JBP proteins prompted the discovery of mammalian ten eleven translocation (TET) enzymes that convert 5-methylcytosine (5mC) to hydroxy-5mC (5hmC) [*T. brucei* JBP proteins converting methyl-U (T) to hmU]. TET enzymes are important factors for reprogramming during development and differentiation (Pastor et al., 2013; Delatte et al., 2014). TET1/2-depleted trophoblast cells showed increased polyploidy due to endoreduplication, defective G2-M transition, and centriole separation (Chrysanthou et al., 2018). Regions adjacent to DNA damage accumulate 5hmC modifications in a TET2-dependent manner in human cells. Mouse ESC lacking TET1–3 enzymes exhibited an abnormal chromosome segregation that was further aggravated by aphidicolin-induced replication stress (Kafer et al., 2016). Different from these phenotypes associated with TET deficiencies, the loss of JBP1 and JBP2 in BF *T. brucei* does not lead to cell-cycle and cell growth defects. More importantly, the PF *T. brucei* does not have a base J modification, indicating that base J is not required for normal cell growth and cell-cycle progression in PF stage. Although base J (and/or JBP1/2 proteins) is required for cell-cycle control in the H3vΔ H4vΔ condition, its cellular and molecular functions are still unclear. In addition to this discrepancy between BF and PF trypanosomes, the base J’s role is more important in transcription termination in *Leishmania* in which JBP1 is essential. This could be due to that *Leishmania* does not have an obvious H4v homolog. The cellular function of site-specific base J in *T. brucei* remains unclear, and further studies are needed to understand its roles, perhaps using non-targeted screening methods, e.g., mass spectrometry, to identify JBP1 or JBP2 interacting proteins or synthetic lethality screening in JΔ trypanosome cells.

H3v, but not H4v, binds telomere repeats (Siegel et al., 2009). Consistent with this, deletion of H3v, but not H4v, disrupts VSG silencing. If canonical H3–H4 nucleosomes occupy telomeres in H3vΔ mutant, the same VSG-silencing phenotype should occur in the H3vΔ and H4vΔ H3vΔ mutants. However, the synergistic VSG-silencing defect observed in Tet-treated DKO indicates that H3vΔ and Tet-treated DKO cells may have different telomeric chromatin structures. One possibility is that H4v nucleosomes occupy telomeres in the H3vΔ mutant. In Tet-treated DKO cells, telomeres may be nucleosome free or occupied by H3–H4 nucleosomes that cannot maintain telomeric heterochromatin. Nucleosome-free telomere DNA may disturb (sub)telomeric heterochromatin structure, which leads to the loss of telomere silencing and activation of DDR (Figure 9B).

## Materials and Methods

### *Trypanosoma brucei* Strains and Plasmids

Bloodstream-form *T. brucei* (the Lister 427 antigenic type MITat1.2 clone 221a) was cultured in HMI-9 at 37°C (Hirumi and Hirumi, 1989). All cell lines were constructed in a “SM” background, which has a tetracycline-inducible system constitutively expressing T7 RNA polymerase and Tet repressor (Wirtz et al., 1999). CRE gene under Tet operator control is stably integrated at an rDNA spacer locus, and its expression can be induced by tetracycline addition.

Genes encoding JBP1, JBP2, H3v, and H4v were deleted using KO vectors. These vectors have gene-specific homology sequence arms that are homologous to upstream or downstream sequences of a target gene. Between the upstream and downstream homology arms, a selection marker flanked by loxP sites is located. A positive selection marker (hygromycin resistance gene, HYG, or puromycin-resistance gene, PUR) conjugated with herpes simplex virus thymidine kinase gene (HSVTK or TK) was used. After deleting both alleles of a gene, tetracycline (Sigma-Aldrich) can be added to express Cre to remove the floxed HYG-TK and PUR-TK markers, so these selection markers can be reused to delete the next gene. To generate a conditional null mutant, a “conditional KO (cKO or floxed)” allele was introduced using a cKO vector, which is the same as the KO vector except that H3v-Ty1, H3v-HA, or Flag-H4v gene is inserted in between of the 5′ loxP and a selection marker gene. Cre can remove a region between two loxP sites (including the H3v-Ty1 or Flag-H4v allele), thereby generating cells that no longer express H3v or H4v gene upon tetracycline addition. WT (HSTB-904), JΔ (HSTB-778), H3vΔ (HSTB-881), and H3vΔ JΔ (HSTB-868) were generated previously (Schulz et al., 2016). In this study, the following strains were generated.

H4vΔ strain: One H4v allele in the wild-type strain (HSTB-904) was deleted with pKP26 vector containing an H4v-KO-HYG-TK cassette (“H4v-KO” indicates that the cassette contains homology arms for H4v deletion), creating an H4vΔ/+ heterozygote (HSTB-1039). The second H4v allele in HSTB-1039 was deleted with pKP27 containing an H4v-KO-PUR-TK, creating an H4vΔ/Δ homozygote (HSTB-1051). Selection markers in HSTB-1051 were removed by Cre expression (HSTB-1067).

H4vΔ JΔ strain: One H4v allele in the JΔ strain (HSTB-778) was deleted with pKP26 (H4v-KO-HYG-TK), creating an H4vΔ/+ JΔ (HSTB-1041). The second H4v allele in HSTB-1041 was deleted with pKP27 (H4v-KO-PUR-TK), creating an H4vΔ/Δ JΔ (HSTB-1055). Selection markers in HSTB-1055 were removed by Cre expression (HSTB-1069).

H4vΔ H3vΔ strain: One H4v allele in the H3vΔ strain (HSTB-881) was deleted with pKP26 (H4v-KO-HYG-TK), creating an H4vΔ/+ H3vΔ (HSTB-1043). The second H4v allele in HSTB-1043 was deleted with pKP27 (H4v-KO-PUR-TK), creating H4vΔ/Δ H3vΔ strains (HSTB-1260, 1261, 1262, 1263, 1264, and 1265).

An attempt to generate H4vΔ H3vΔ JΔ strain: One H4v allele in the H3vΔ JΔ strain (HSTB-868) was deleted with pKP26 (H4v-KO-HYG-TK), creating H4vΔ/+ H3vΔ JΔ (HSTB-1045). pKP27 (H4v-KO-PUR-TK) was transfected in HSTB-1045. Clones were obtained initially but did not expand further.

Floxed H3v-Ty1 in H4vΔ strain (DKO with a floxed H3v-Ty1): One H3v allele was deleted in the H4vΔ strain (HSTB-1067) with pDS88 (H3v-KO-HYG-TK), creating an H3vΔ/+ H4vΔ strain (HSTB-1074). Cre was induced to remove the HYG-TK selection marker (HSTB-1078). The remaining H3v allele in the H3vΔ/+ H4vΔ strain (HSTB-1078) was replaced with a floxed H3v-Ty1-PUR (pKP33 vector), creating a floxed H3v-Ty1/Δ H4vΔ strain (HSTB-1082, DKO). The H3v-Ty1 allele can be removed by tetracycline addition, generating H3vΔ H4vΔ double-KO cells (Tet-treated DKO).

Floxed H3v-Ty1 in H4vΔ JΔ strain (TKO with a floxed H3v-Ty1): One H3v allele was deleted in the H4vΔ JΔ strain (HSTB-1069) with pDS88 (H3v-KO-HYG-TK), creating an H3vΔ/+ H4vΔ JΔ strain (HSTB-1076). Cre was induced to remove a HYG-TK selection marker (HSTB-1080). The remaining H3v allele in the H3vΔ/+ H4vΔ JΔ strain (HSTB-1080) was replaced with a floxed H3v-Ty1-PUR (pKP33 vector), creating a floxed H3v-Ty1/Δ H4vΔ JΔ strain (HSTB-1088, TKO). The H3v-Ty1 allele can be removed by tetracycline addition, generating H3vΔ H4vΔ JΔ triple-KO cells (Tet-treated TKO).

Floxed H3v-HA in H4vΔ JΔ strain (TKO with a floxed H3v-HA): The remaining H3v allele in the H3vΔ/+ H4vΔ JΔ strain (HSTB-1080) was replaced with a floxed H3v-HA-PUR-TK (pDS84 vector), creating a floxed H3v-HA/Δ H4vΔ JΔ strain (HSTB-1142). The H3v-HA allele can be removed by tetracycline addition, generating H3vΔ H4vΔ JΔ triple-KO cells.

Floxed Flag-H4v in H3vΔ JΔ strain (TKO with a floxed Flag-H4v): The remaining wild-type H4v allele in the H4vΔ/+ H3vΔ JΔ strain (HSTB-1045) was replaced with a floxed Flag-H4v-PUR-TK (pKP49), creating a floxed Flag-H4v/Δ H3vΔ JΔ strain (HSTB-1098). The Flag-H4v allele can be removed by tetracycline addition, generating H4vΔ H3vΔ JΔ triple-KO cells.

Triple KO strain expressing HA-tagged *Tb*RPA1 protein: One allele of RPA1 was epitope tagged with 3xHA by one-step PCR integration using a pMOTag-3H vector (Oberholzer et al., 2006), in the floxed H3v-Ty1 H4vΔ JΔ strain (HSTB-1088), generating HSTB-1093.

Bloodstream-form *T. brucei* strains were maintained in HMI-9 media containing necessary antibiotics at the following concentrations: 2.5 μg/ml of G418 (Sigma-Aldrich), 5 μg/ml blasticidin (InvivoGen), 1 μg/ml phleomycin (InvivoGen), 5 μg/ml hygromycin (InvivoGen), 0.1 μg/ml puromycin (InvivoGen), 1 μg/ml tetracycline (Sigma-Aldrich), and 35 μg/ml ganciclovir (Sigma-Aldrich). *T. brucei* strains, plasmids, and oligonucleotides used in this study are listed in Supplementary Table 1.

### Western Blot

Five to 10 million cells were collected and suspended in the Laemmli buffer and separated on an SDS-PAGE gel (Bio-Rad). Following transfer to nitrocellulose membrane (GE Healthcare), the proteins were analyzed using mouse anti-Ty1, mouse anti-HA (Sigma-Aldrich), mouse-anti-Flag (Sigma-Aldrich), mouse anti-VSG3 (Antibody and Bioresource Core Facility, MSKCC), rabbit anti-VSG3, rabbit H3 (Abcam), or mouse anti-tubulin (Sherwin and Gull, 1989) antibodies.

### Flow Cytometry Analysis for Cell-Cycle, BrdU Pulse, and Variant Surface Glycoprotein Switching Experiments

About 10 million cells of WT, DKO, and TKO strains treated or untreated with tetracycline were collected and fixed with ice-cold 70% ethanol. To stain DNA, fixed cells were incubated with 50 μg/ml PI (Sigma-Aldrich) and 200 μg/ml RNase A (Sigma-Aldrich) in phosphate-buffered saline (PBS; Corning) at 37°C for 30 min. Cell-cycle progression was analyzed using LSR II or Via (BD Biosciences), FACSdiva software (BD Biosciences), and FlowJo software package (FlowJo).

Bromo-26deoxyuridine pulse experiments were performed as described previously (Ryba et al., 2011), with some modifications (Kim, 2019). BrdU (Sigma-Aldrich) was added to a final concentration of 500 μM to cell cultures and incubated for 40 min. Cells were then fixed with ice-cold 70% ethanol, incubated in 0.1 N HCl (Fisher Scientific) with 0.5% Triton X-100 (Fisher Scientific), and washed with PBS containing 0.5% Tween 20 and 1% BSA (Jackson ImmunoResearch). To detect BrdU incorporation, fixed cells were incubated with 1 μg/ml mouse anti-BrdU antibody (Fisher Scientific; BD Pharmingen) at room temperature for 2 h and incubated with donkey anti-mouse-Alexa 488 (Invitrogen Molecular Probes) at 4 μg/ml for 45 min at room temperature in the dark. Cells were incubated in PBS containing 5 μg/ml PI and 250 μg/ml RNase A for 30 min at room temperature in the dark and then analyzed by flow cytometry using LSR II or Via (BD Biosciences), FACSdiva software (BD Biosciences), and FlowJo software package (FlowJo).

Variant Surface Glycoprotein switching from VSG2 to VSG3 was examined by staining cells with antibodies conjugated with different fluorophores. VSG2-expressing cells containing 0, 0.01, 0.1, 1, or 10% of VSG3-expressing cells and Tet-untreated or treated WT, DKO, and TKO cells were prepared. Six million live cells were stained with mouse anti-VSG2 antibody (Antibody and Bioresource Core Facility, MSKCC) conjugated with Dylight 488 (Abcam) and mouse anti-VSG3 antibody (Antibody and Bioresource Core Facility, MSKCC) conjugated with Dylight 650 (Abcam). One million cells were analyzed with Via (BD Biosciences).

### Stranded RNA-Seq With rRNA Depletion

WT, KO mutants, Tet-untreated DKO and TKO, and Tet-treated (for 2 days) DKO and TKO cells in triplicate, three DS clones, and three TS clones were prepared. About 50 million cells were collected, and total RNA was extracted using the RNA STAT-60 (Tel-Test) according to the manufacturer’s protocol, quantified on a NanoDrop2000c, and further cleaned using RNeasy Kit (Qiagen). rRNA was removed using the Ribo-Zero kit (Illumina), and stranded RNA-seq libraries were prepared using random hexamer and NEB Directional RNA Library Prep Kit (NEB) and sequenced on the NovaSeq 6000 PE150.

Read quality was analyzed using the FastQC program, and reads were trimmed using the Trim Galore program from Galaxy^1^ (parameters: phred 20, stringency 1 bp, error 0.1, and length 20), aligned with Bowtie 2 (Langmead and Salzberg, 2012) to the Lister 427 (Müller et al., 2018), *Tb*927v5^2^, or VSGnome (Cross et al., 2014), and analyzed using SeqMonk algorithm^3^ from Babraham Bioinformatics.

To examine transcription profiles at chromosome level, each chromosome was binned at 5-kb resolution with 1-kb step (or 10-kb bin, 2.5-kb step for analyses of DS and TS clones) as described previously (Kim, 2019). RPM values from forward only or reverse reads only were generated (Supplementary Tables 3, 9), and fold changes between WT and mutant were analyzed. To compare gene expression between WT and mutants, reads mapping to 8,428 CDSs were analyzed. RPKM values were generated from all reads, reads mapping to opposite direction of genes (sense transcription), or reads mapping the same direction as genes (antisense transcription; Supplementary Table 4). To examine VSG expression, trimmed reads were aligned to the VSGnome^4^ (Cross et al., 2014). RPKM values (Supplementary Table 6) were displayed in box plots. Alignment report, correlation between replicates, and PCA are summarized in Supplementary Table 2 and Supplementary Figure 4.

### Stranded RNA-Seq With polyA Selection

WT, JΔ, H4vΔ, H4vΔ JΔ, and Tet-treated or untreated TKO strains were grown in triplicate. About 50 million cells were collected, and total RNA was extracted using the RNA STAT-60 (Tel-Test) according to the manufacturer’s protocol, quantified on a NanoDrop2000c, and further cleaned using RNeasy kit (Qiagen). RNA-seq libraries were prepared from 500 ng of RNA samples using an oligo dT-based method with 15 cycles of PCR amplification with the Illumina TruSeq mRNA stranded kit (Illumina) and sequenced on the Illumina HiSeq 2000 v4 (50-bp single-end read). Reads were analyzed for transcription profile (Supplementary Table 5) and gene expression as above (Supplementary Table 4). Alignment report, correlation between replicates, and PCA are summarized in Supplementary Table 2 and Supplementary Figure 4.

### MFA-Seq

MFA-seq was performed as reported previously (Tiengwe et al., 2012), with some modifications (Kim, 2019). Floxed H3v-HA TKO cells (HSTB-1142) were treated with tetracycline for 0, 1, and 2 days. About 100 million cells at ∼1 × 10^6^/ml density were fixed with ice-cold 70% ethanol and DNA stained with 50 μg/ml PI in PBS. Cells in G1, early S, late S, and G2 phases were FACS-sorted (BD Aria3: BD Biosciences), and genomic DNA was isolated using the QIAamp DNA Blood Mini Kit (Qiagen). About 10 ng of genomic DNA was fragmented using the NEBNext dsDNA Fragmentase (NEB), and sequencing libraries were generated using the NEBNext Ultra DNA Library Prep Kit (NEB) and NEB Multiplex Oligos (NEB) for Illumina according to the manufacturer’s protocol. Sequencing was performed on an Illumina HiSeq 2000 sequencer (50-bp single-end read). Read quality was analyzed as above, quality checked using FastQC, trimmed using Trim galore, and aligned with Bowtie 2 to the Lister 427 genome. About 3–15 million reads were analyzed using the SeqMonk algorithm. To examine replication initiation profiles, chromosomes were binned at 10-kb resolution with 2.5-kb step and RPM values were generated with SeqMonk (Supplementary Table 8). RPM values from the S phase samples were normalized to values from G1 phase and plotted.

### Replication Protein A1 Immunofluorescence

Floxed H3v-Ty1 TKO strain expressing *Tb*RPA1 tagged with HA (HSTB-1093) was treated with tetracycline for 0, 1, and 2 days. Cells were fixed with 0.5% paraformaldehyde (Thermo Fisher Scientific, Pierce) for 10 min, permeabilized with 0.2% NP-40 (Sigma-Aldrich) in PBS, and incubated with mouse anti-HA antibody and then with the secondary antibody conjugated with Donkey-anti-mouse Alexa 567 (Invitrogen Molecular Probes). DNA was stained with 0.5 mg/ml DAPI (Sigma-Aldrich). Images were captured using a Zeiss Axioplan 2 fluorescence microscope and edited with Adobe Photoshop.

## Data Availability Statement

The datasets generated in this study can be found in online repositories. The names of the repository/repositories and accession number(s) can be found in https://www.ncbi.nlm.nih.gov/sra (accession number, PRJNA727846).

## Author Contributions

H-SK designed the study, performed the experiments, analyzed data, wrote and revised the manuscript, and submitted the manuscript.

## Conflict of Interest

The author declares that the research was conducted in the absence of any commercial or financial relationships that could be construed as a potential conflict of interest.

## Publisher’s Note

All claims expressed in this article are solely those of the authors and do not necessarily represent those of their affiliated organizations, or those of the publisher, the editors and the reviewers. Any product that may be evaluated in this article, or claim that may be made by its manufacturer, is not guaranteed or endorsed by the publisher.

## Footnotes

1
https://usegalaxy.org

2
www.tritrypdb.org

3
http://www.bioinformatics.babraham.ac.uk/projects/seqmonk/

4 Retrieved from http://tryps.rockefeller.edu
