# Supplementary material for: Genetic Interaction Between Site-Specific Epigenetic Marks and Roles of H4v in Transcription Termination in Trypanosoma brucei
Source: Front Cell Dev Biol. 2021 Oct 14;9:744878. doi: 10.3389/fcell.2021.744878 (PMC8551723; doi:10.3389/fcell.2021.744878)
Supplement: Supplementary file 1 [file Data_Sheet_1.PDF]

Supplementary Information

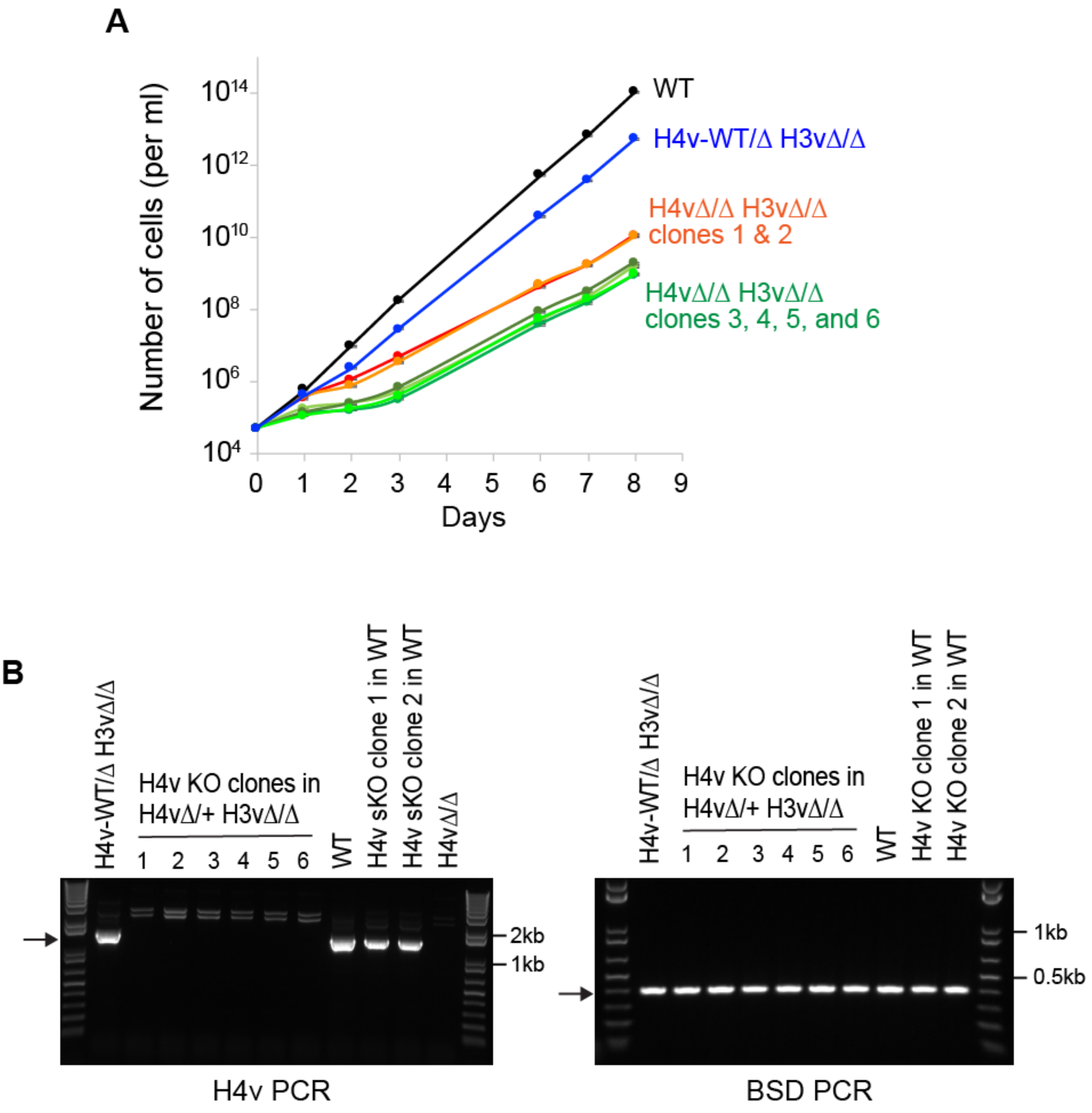

Supplementary Figure 1. Growth of H3vΔ H4vΔ cell lines generated by stable transfection of a H4v KO cassette (linearized pKP27 plasmid) in H4vΔ/+ heterozygote H3vΔ strain. (A) Cell growth: WT and 6 clones of H3vΔ H4vΔ double mutant, and the parental H4v+/Δ H3vΔ/Δ strain. **(B)** PCR genotyping of H4v allele. BSD PCR is shown as a control.

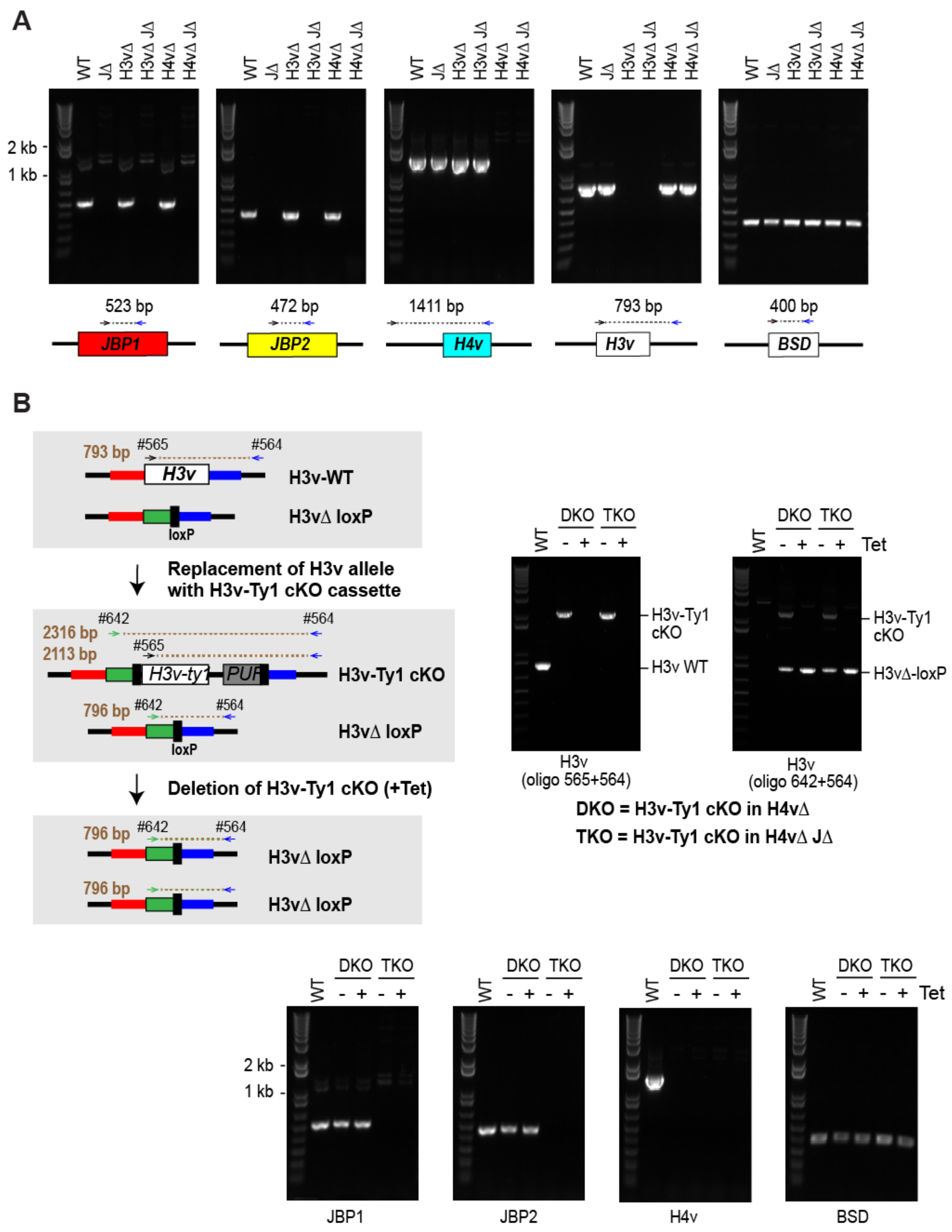

Supplementary Figure 2. PCR genotyping of KO mutants and conditional KO mutants (DKO and TKO). **(A)** PCR genotyping for JBP1, JBP2, H3v, and/or H4v KO **(B)** PCR genotyping confirms the removal of the floxed H3v-Ty1 allele 2 days after tetracycline treatment in DKO and TKO strains. H4v, JBP1 and JBP2 were also PCR genotyped in these strains. Diagram showing PCR genotyping strategy for conditional H3v-Ty1 KO.

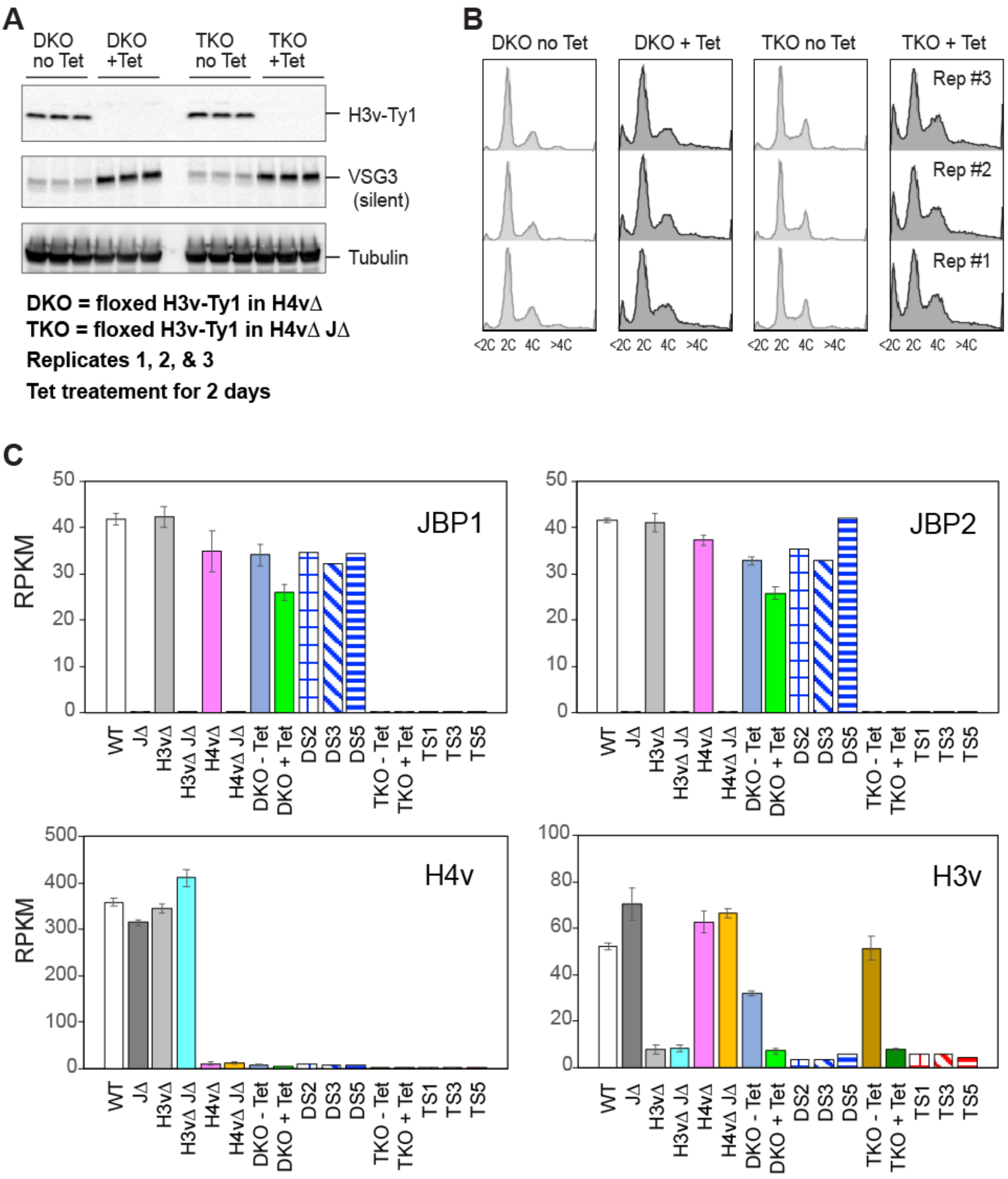

Supplementary Figure 3. H3v-Ty1 removal in DKO and TKO strains with or without tetracycline in triplicate (samples used in rRNA-depleted stranded RNA-seq). (A) Removal of H3v-Ty1 protein in Tet-treated DKO and TKO cells by western blot. **(B)** Cell-cycle profile by flow cytometry **(C)** Levels of H3v, H4v, JBP1 and JBP2 RNA in the KO mutants, DS, and TS clones used in rRNA-depleted stranded RNA-seq. RPKM values for JBP1, JBP2, H4v and H3v CDS in WT and KO mutants, DS and TS clones.

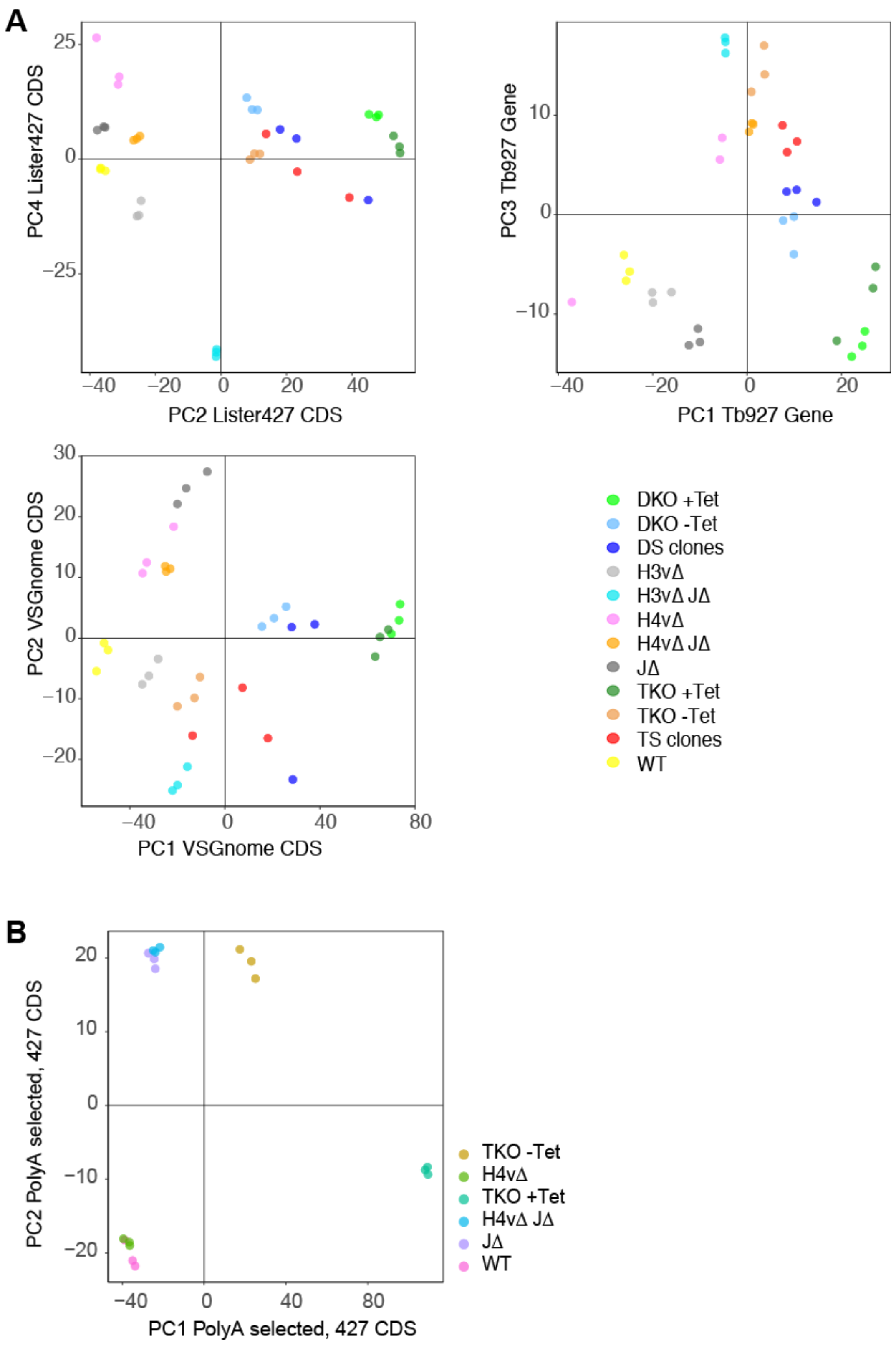

Supplementary Figure 4. (A) PCA plots obtained from triplicated samples used for rRNA-depleted stranded RNA-seq. Sequence reads were mapped to CDSs in the Lister 427 genome, genes in the *Tb*927v5 genome, and CDSs in the VSGnome. **(B)** PCA plot obtained from triplicated samples used for polyA-selected stranded RNA-seq. Sequence reads were mapped to CDSs in the Lister 427 genome.

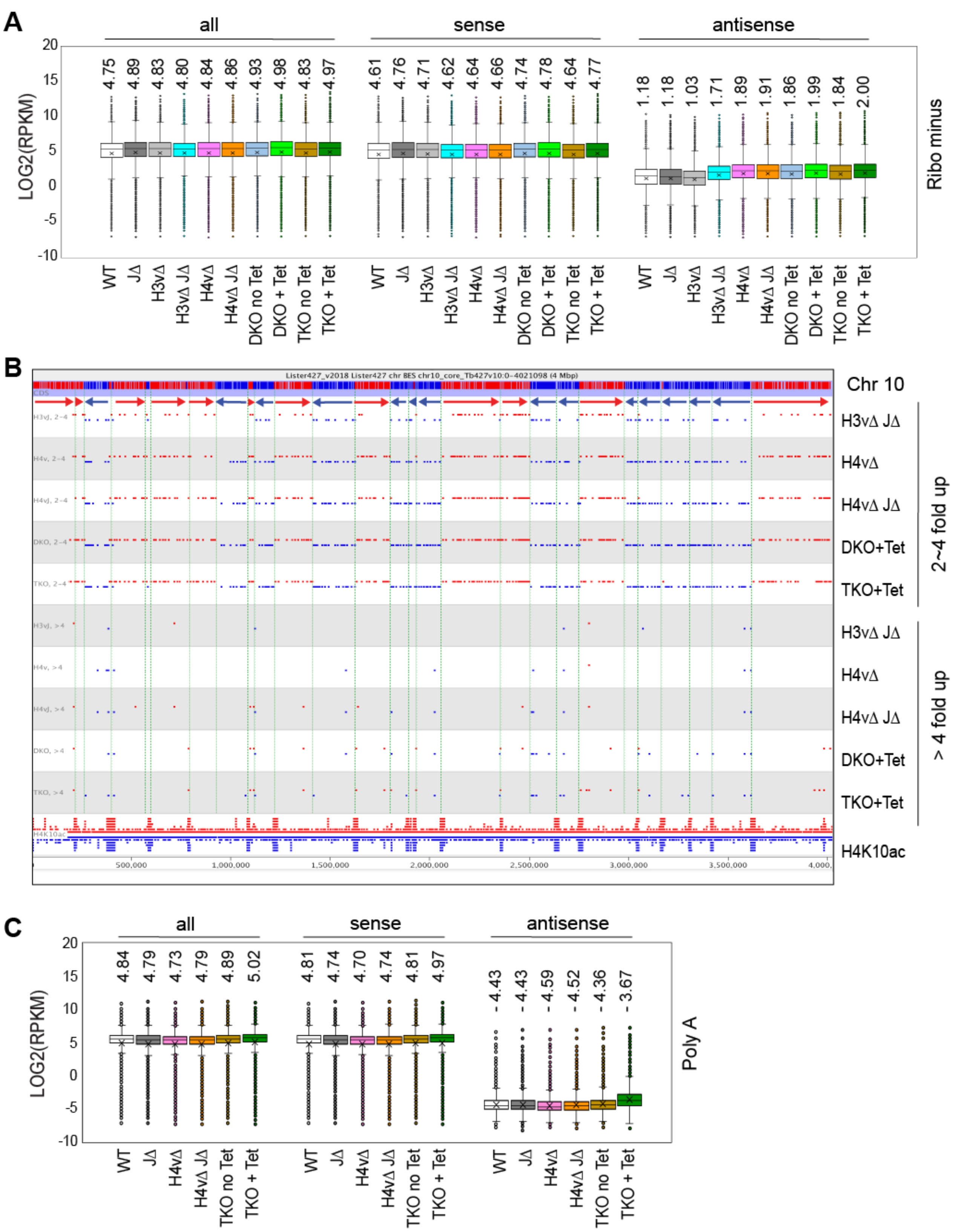

Supplementary Figure 5. Increased antisense transcription levels in H4vΔ mutants. (A and C) Log2(RPKM) values from all reads, reads mapping to opposite direction of genes (sense transcription) or reads mapping the same direction as genes (antisense transcription) were obtained for 8,428 CDSs. Box plots compare between wild type and KO mutants (A: data from rRNA-depleted stranded RNA-seq, C: polyA-selected stranded RNAseq). Mean values are shown and indicated as x. Outliers are shown as circles. Statistical analysis is summarized in Supplementary Table 7. **(B)** Location of CDSs in chromosome 10 that were 2~4-fold upregulated or more than 4-fold upregulated in H3vΔ JΔ, H4vΔ, H4vΔ JΔ, and Tet-treated DKO and TKO, compared to WT.

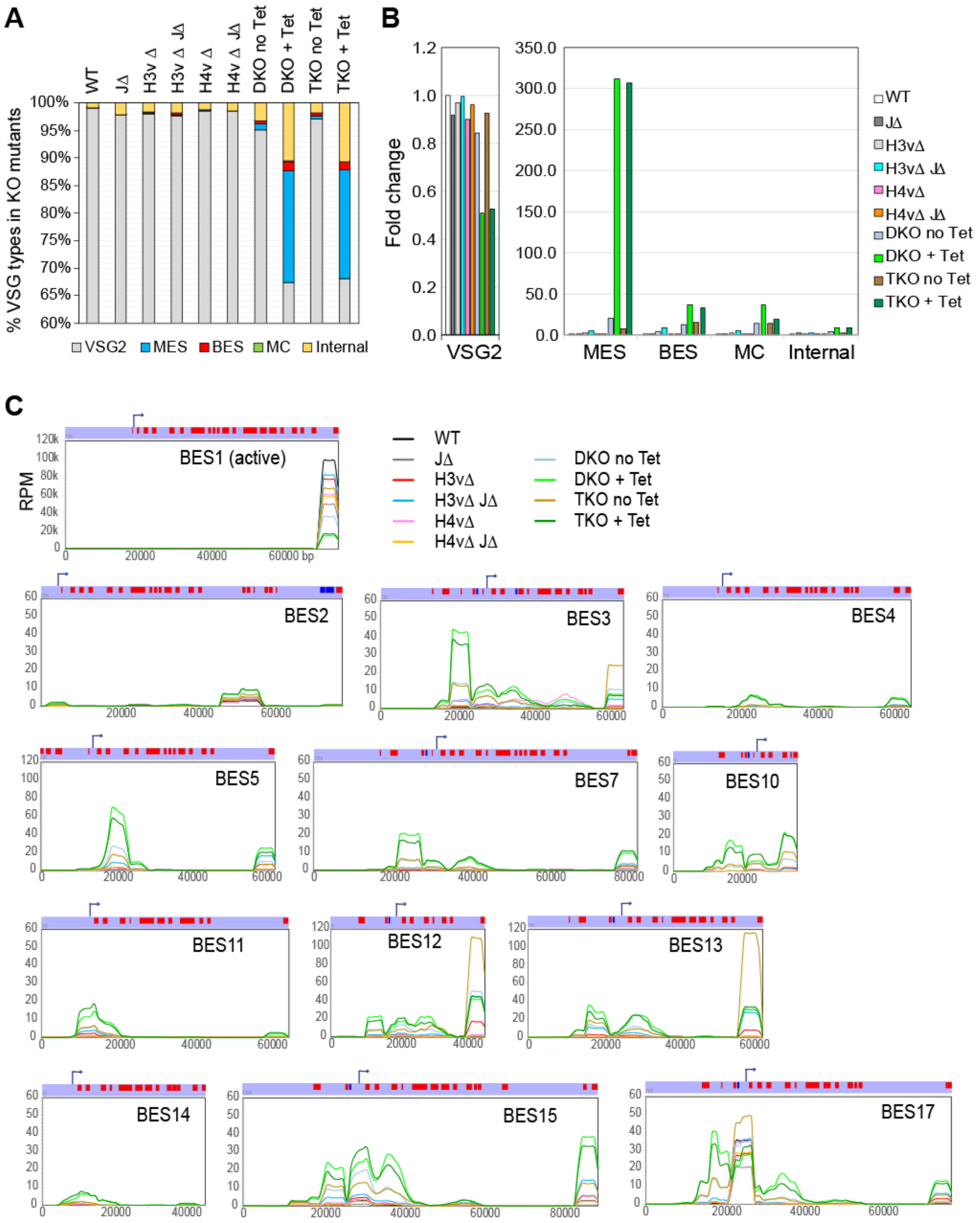

Supplementary Figure 6. TTS chromatin marks are important in repression of promoter proximal and telomere proximal genes within BESs. (A) Percentage of the active VSG2 and each silent VSG type, including BES, MES, minichromosomal (MC) and chromosome internal (Internal) VSGs. **(B)** Fold changes of each VSG type between KO mutant and WT. **(C)** Derepression of silent BES transcription units. Lister 427

reference genome also contain BES sequence information. RPM values from reads mapping to BESs were plotted over each BES. Location of RNA pol I promoter is shown as arrows with bent tips.

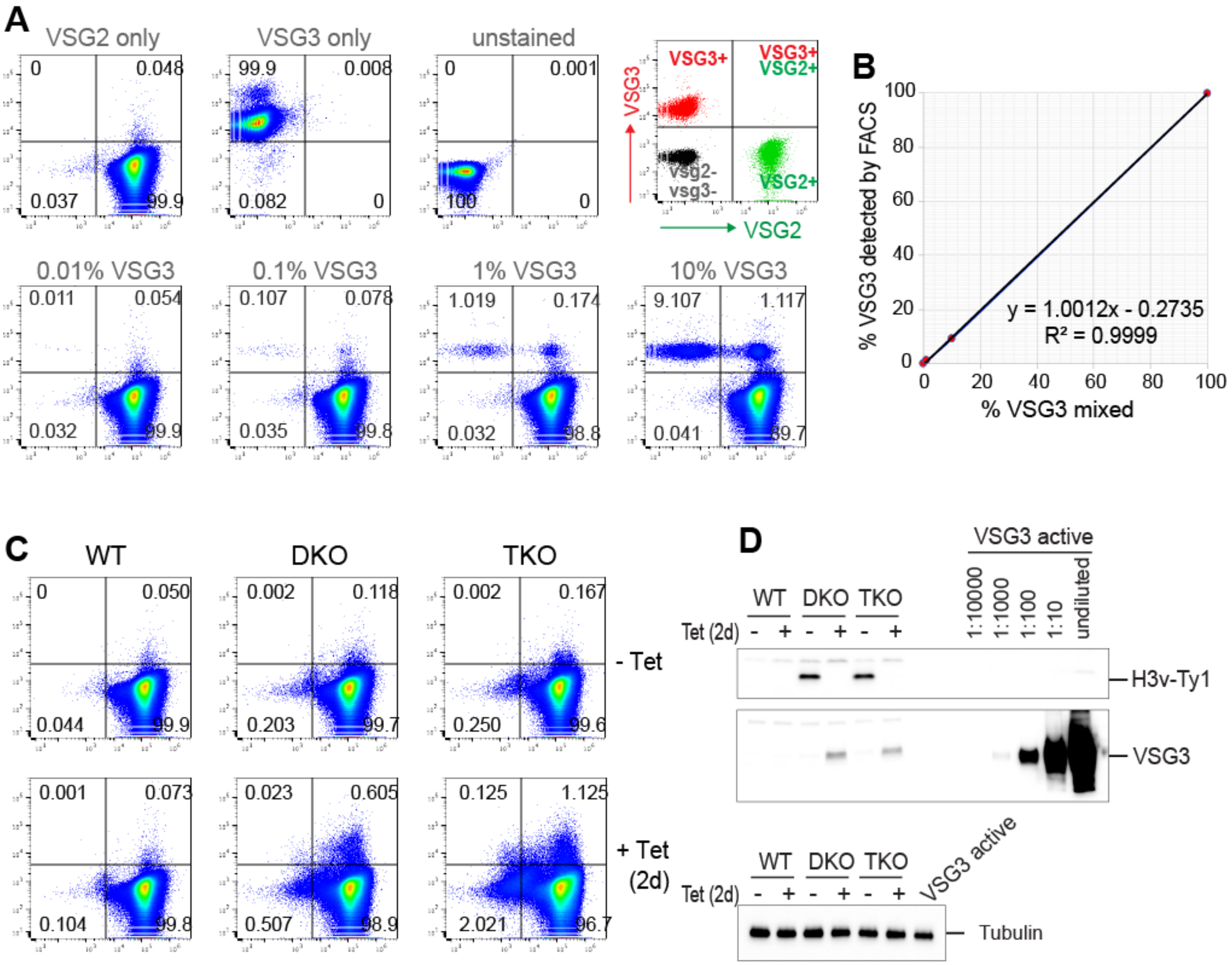

Supplementary Figure 7. VSG switching in DKO and TKO strains. (A) Detection of non-VSG2 expressing cells by flow cytometry. VSG2 expressing cells were mixed with VSG3 expressing cells at an indicated percentage. Live cells expressing VSG2 alone or VSG3 alone, or both were incubated with fluorophore conjugated VSG antibodies, anti-VSG2-Dylight 488 and anti-VSG2-Dylight 650, and then analyzed by flow cytometry. **(B)** Percent of VSG3 mixed in and percent of VSG3 detected by FACS. **(C)** WT, DKO and TKO cells treated with or without Tet were incubated with anti-VSG2-Dylight 488 and anti-VSG2-Dylight 650, and then analyzed by flow cytometry. **(D)** Whole cells from (C) were collected and analyzed by western blot.

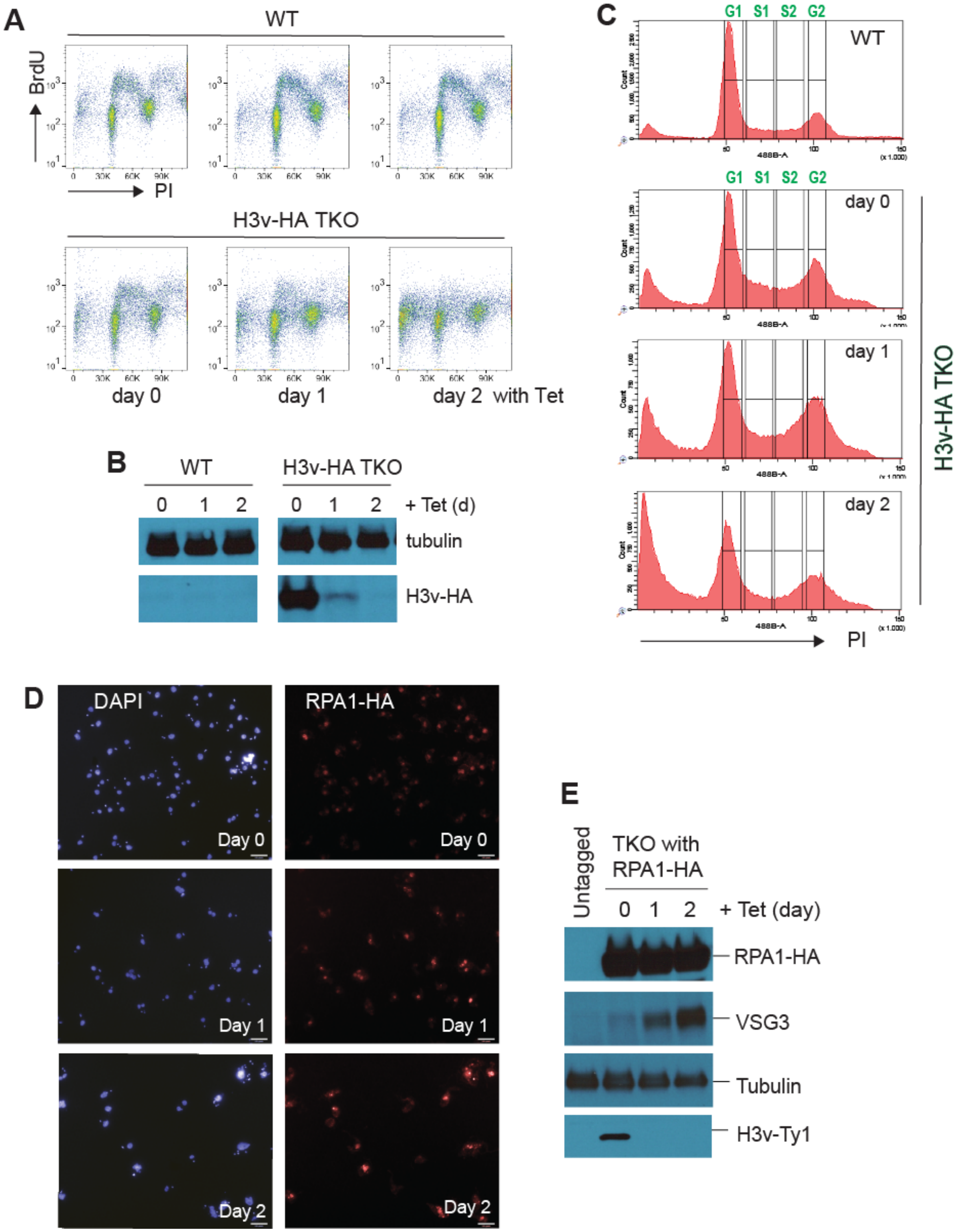

Supplementary Figure 8. Control data for Tet-induced TKO prepared for MFA-seq experiment and RPA1 nuclear foci formation in Tet-induced TKO. (A) BrdU incorporation assay. WT (Kim, 2019) and floxed H3v-HA TKO strain treated with tetracycline for 0, 1, and 2 days were pulse-labeled with 500 µM BrdU for 40 min. Bulk DNA was stained with PI, and cells were analyzed by flow cytometry. **(B)** Western blot confirming depletion of H3v-HA in the TKO strain after Tet treatment. Tubulin was used as a loading control. **(C)** FACS sorting for MFA-seq assay; WT and TKO strain treated with Tet for 0, 1, and 2 days. **(D & E)** Formation of RPA1 nuclear foci in TKO cells. Tet-treated TKO cells expressing *Tb*RPA1-HA were fixed and examined by immunofluorescence. Bulk DNA was stained with DAPI (blue) and *Tb*RPA1 with mouse anti-HA followed by secondary antibodies conjugated with Alexa 567(red). Proteins were analyzed by western blot.

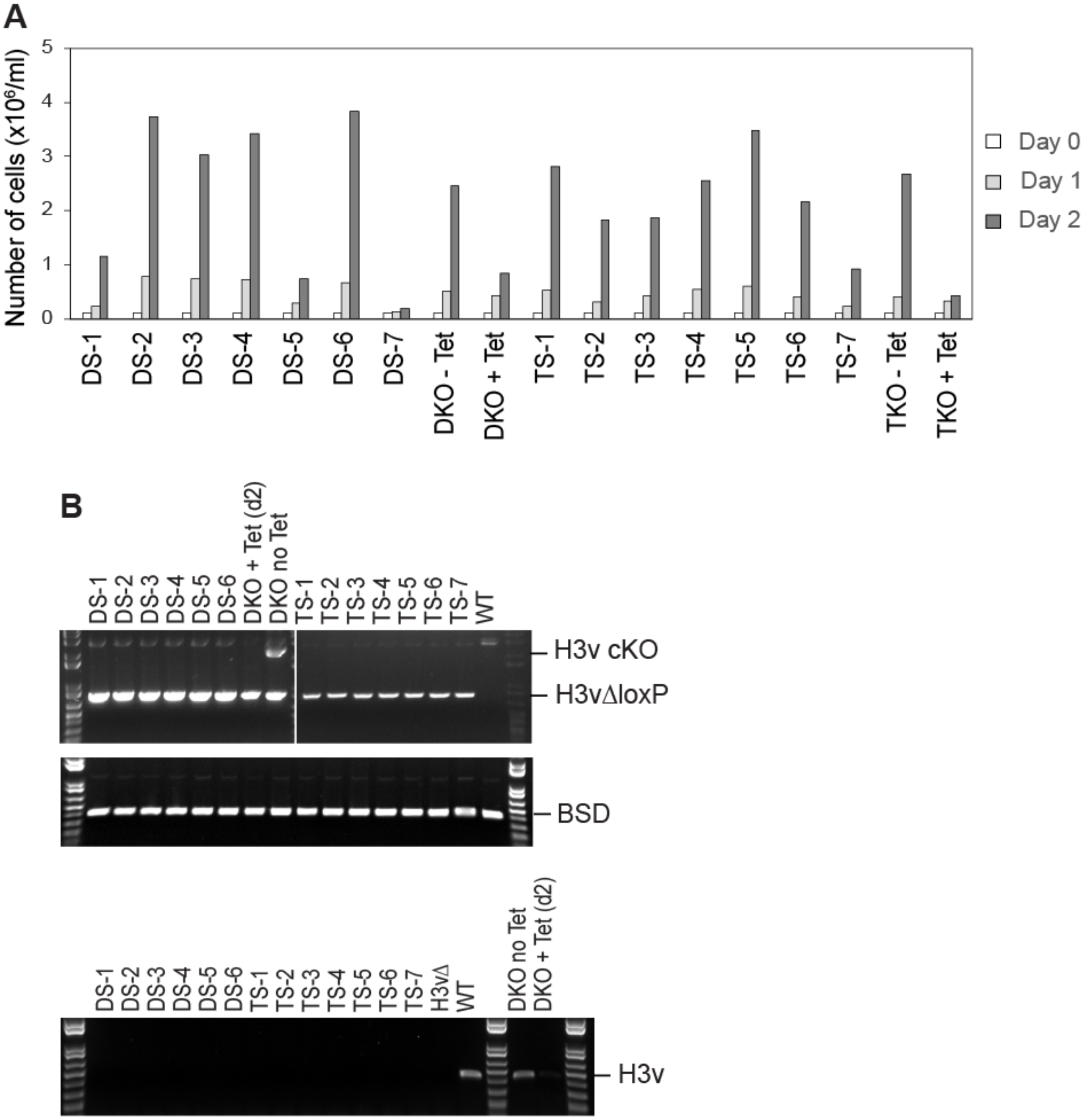

Supplementary Figure 9. Control data for DS and TS clones. (A) Growth of DS and TS clones. Surviving clones identified from the 96-well plate (7 DS and 7 TS clones) were monitored for cell growth. **(B)** PCR genotyping confirming the loss of H3v-Ty1 gene.

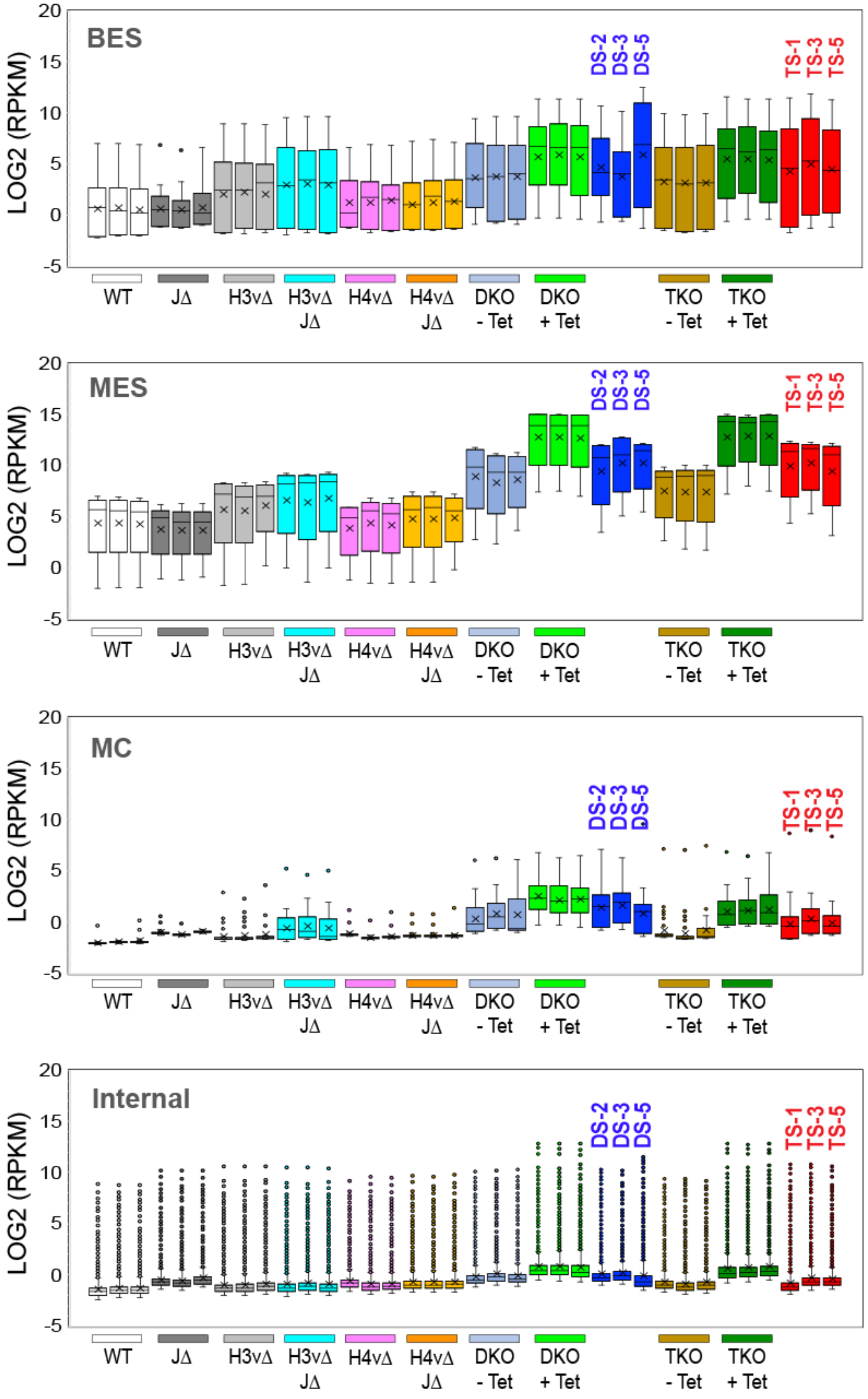

Supplementary Figure 10. Expression of BES, MES, minichromosomal (MC) and chromosome-internal VSGs in DS and TS clones was compared with replicates of WT and KO mutant strains.

Supplementary Tables

Supplementary Table 1. List of cell lines, plasmids and oligo nucleotides used in this study

Supplementary Table 2. Summary of RNAseq and MFAseq analysis-Bowtie2 mapping, correlation between replicates, and PCA analysis

Supplementary Table 3. rRNA-depleted stranded RNA-seq mapped to Lister 427 and analyzed with sliding window (chromosome core and BESs)

Supplementary Table 4. Transcriptome analyses

Supplementary Table 5. PolyA-selected stranded RNA-seq mapped to Lister 427 and analyzed with sliding window (chromosome core and BESs)

Supplementary Table 6. VSGnome mapping

Supplementary Table 7. Statistics for transcriptome analysis and VSGs

Supplementary Table 8. MFA-seq mapped to Lister 427 and analyzed with sliding window (10kb bin, 2.5kb step, RPM value)

Supplementary Table 9. rRNA-depleted stranded RNA-seq mapped to Lister 427 and analyzed with sliding window for DS and TS clones
